# Complex Conformational Space of the RNA Polymerase II C-Terminal Domain upon Phosphorylation

**DOI:** 10.1021/acs.jpcb.3c02655

**Published:** 2023-10-23

**Authors:** Weththasinghage D. Amith, Bercem Dutagaci

**Affiliations:** Department of Molecular and Cell Biology, University of California, Merced, Merced, California 95343, United States

## Abstract

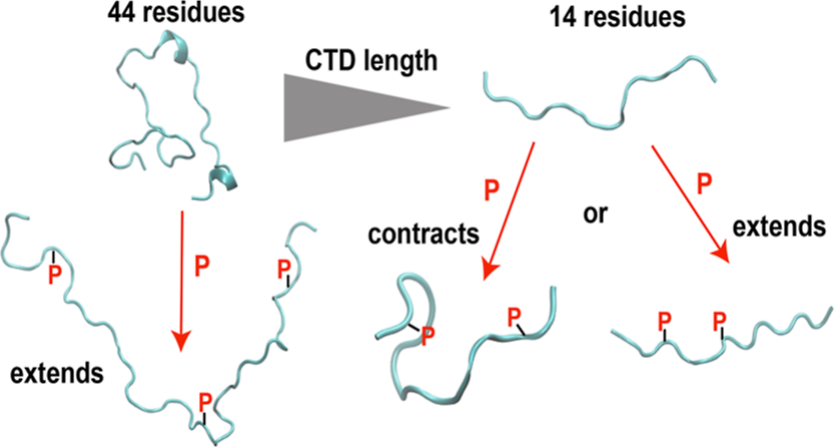

Intrinsically disordered proteins (IDPs) have been closely studied during the past decade due to their importance in many biological processes. The disordered nature of this group of proteins makes it difficult to observe its full span of the conformational space using either experimental or computational studies. In this article, we explored the conformational space of the C-terminal domain (CTD) of RNA polymerase II (Pol II), which is also an intrinsically disordered low complexity domain, using enhanced sampling methods. We provided a detailed conformational analysis of model systems of CTD with different lengths; first with the last 44 residues of the human CTD sequence and finally the CTD model with 2-heptapeptide repeating units. We then investigated the effects of phosphorylation on CTD conformations by performing simulations at different phosphorylated states. We obtained broad conformational spaces in nonphosphorylated CTD models, and phosphorylation has complex effects on the conformations of the CTD. These complex effects depend on the length of the CTD, spacing between the multiple phosphorylation sites, ion coordination, and interactions with the nearby residues.

## Introduction

During the last decades, intrinsically disordered proteins (IDPs) have been recognized as an important class of proteins due to their relevance to many biological processes.^1,2^ Cell signaling and regulation,^3^ stress response,^4^ human neurodegenerative diseases,^5^ and cellular liquid–liquid phase separation (LLPS)^6,7^ are some of the biological phenomena which are associated with IDPs. One of the most challenging aspects of IDPs is to determine the full span of their conformational space using currently available experimental techniques such as small-angle X-ray scattering (SAXS), nuclear magnetic resonance (NMR) spectroscopy, and circular dichroism (CD) spectroscopy. Such experimental methods have provided information on the structural and dynamic features of IDPs, such as backbone conformations, secondary structures, overall shape, and size of the molecules.^8–10^ However, converting these features to actual conformational ensembles remains a challenge. Therefore, the common approach became to develop and apply computational methods to obtain conformational spaces of IDPs and to use experimental features for assisting and validating the computational models. These computational methods include Monte Carlo approaches to generate ensembles,^11,12^ approaches based on molecular dynamics (MD) simulations by applying fragmentation of long IDP sequences,^13,14^ or enhanced sampling simulations in atomic or coarse-grained details.^5,15–18^ Generative machine learning models were also developed, which utilize conformations obtained from MD simulations for training.^19–21^ Among computational methods, MD simulations with enhanced sampling techniques appeared to be a powerful method, especially for relatively short sequences, to provide valuable insights into atomic-level interactions of IDPs in order to obtain a broad conformational space.^15,18,22–25^

The C-terminal domain (CTD) of RNA polymerase II (Pol II) is a low complexity domain that contains heptapeptide (YSPTSPS) repeating sequence, with the number of repeating units differing according to the organism.^26^ Pol II CTD is also recognized as an IDP due to its lack of a defined secondary structure.^26^ Also, recent studies have shown Pol II’s involvement in LLPS formation^27,28^ and suggested that CTD may play a fundamental role in such phase separation events. The length and phosphorylation pattern of CTD are also shown to have effects on LLPS formation.^27,28^ Therefore, determination of conformational spaces for CTD upon phosphorylation is significantly important to recognize the structural features that would impact the Pol II CTD-related LLPS inside a cell. Our knowledge on conformational analysis of CTD and structural effects of phosphorylation is limited as there are only a few experimental^26,29–33^ and computational^22,34,35^ studies on the structure of CTD. Hence, in this work, we studied the conformational landscape of model systems of CTD and the effects of phosphorylation on the conformations. We analyzed the CTD models using an enhanced sampling method, replica-exchange molecular dynamics (REMD)^36^ simulations, in order to sample a wide range of probable conformations. We applied REMD simulations on two model systems; one was the 44-residue tail of human CTD, which has available experimental data to validate our simulations,^29^ and the other was a peptide with a sequence of 2-heptapeptide repeats of CTD (2CTD). Simulations were performed on nonphosphorylated and phosphorylated CTD sequences to observe the effects of phosphorylation pattern on the conformations of CTD models. Phosphorylation introduced conformational changes in both CTD models with 44 residues and 2CTD compared to their nonphosphorylated states; however, the phosphorylated models of 2CTD showed complex effects on their conformational space, while 44-residue models showed somewhat expected conformational changes depending on their net charge, which we elaborate in detail under the Results and Discussion section below.

## Computational Methods

### System Preparation and Equilibration

We used two CTD models in this study. The first one is the CTD of the Rpb1 domain of Pol II between the residues 1927 and 1970, which was characterized by earlier computational^22,29^ and experimental^29^ studies, and the experimental NMR data was reported in the Biological Magnetic Resonance Database with accession number of 27063.^29^ The second system is a 14-residue CTD model referred as 2CTD. We additionally modeled four 44-residue and nine 14-residue phosphorylated systems that the sequences were provided in Table 1. Initial structures of CTDs were generated using the CHARMM^37^ package in conjunction with the Multiscale Modeling Tools in Structural Biology (MMTSB) toolset^38^ and MODELER program.^39^ For the 2CTD, first, the CHARMM package was utilized to generate initial coordinates of the first seven residues. Then the rest of the peptide was modeled by the MODELER program, which generated five different models of the same CTD sequence, and we selected the most extended model. For the 44-residue system, we generated coordinates of the first 43 residues using the CHARMM initial coordinate table as it was providing an already extended structure and modeled the last residue using MODELER. Then the solvated systems were prepared using the CHARMM-GUI server^40–42^ with the most extended initial model structures of CTDs from the previous step. Phosphorylation sites were introduced by CHARMM-GUI. For all CTD sequences, the N-terminus and C-terminus were capped with acetyl (ACE) and -NHCH3 (CT3) groups, respectively. Table 1 shows all of the CTD sequences prepared for this study.

**Table 1:** CTD Sequences, Their Net Charges, Number of Residues, and Abbreviations

net charge	CTD sequence	number of residues	abbreviation
–4	SPTYSPTSPKGSTYSPTSPGYSPTSPTYSLTSPAISPDDSDEEN	44	exp-CTD-nonphos
–8	SPTYSPTSPKGSTYSPTSPGYSPTSPTYSLTSPAISPDDSDEEN	44	exp-CTD-5P-40P
–12	SPTYSPTSPKGSTYSPTSPGYSPTSPTYSLTSPAISPDDSDEEN	44	exp-CTD-5P-12P-18P–32P
–10	SPTYSPTSPKGSTYSPTSPGYSPTSPTYSLTSPAISPDDSDEEN	44	exp-CTD-5P-22P-40P
–16	SPTYSPTSPKGSTYSPTSPGYSPTSPTYSLTSPAISPDDSDEEN	44	exp-CTD-5P-12P-18P-25P-32P-40P
0	YSPTSPSYSPTSPS	14	2CTD-nonphos
–2	YSPTSPSYSPTSPS	14	2CTD-2P
–4	YSPTSPSYSPTSPS	14	2CTD-2P-5P
–4	YSPTSPSYSPTSPS	14	2CTD-2P-12P
–6	YSPTSPSYSPTSPS	14	2CTD-2P-5P-12P
–6	YSPTSPSYSPTSPS	14	2CTD-2P-5P-9P
–8	YSPTSPSYSPTSPS	14	2CTD-2P-5P-9P-12P
–4	YSPTSPSYSPTSPS	14	2CTD-2P-9P
–2	YSPTSPSYSPTSPS	14	2CTD-5P
–4	YSPTSPSYSPTSPS	14	2CTD-5P-12P

The phosphorylated serine residues are underlined in the sequence. The CTD sequence with 44 residues from the human CTD of Pol II (residues between 1927 and 1970) and the CTD sequence with 14 residues that contain two repeats of the heptapeptide sequence were selected. In addition, we explored four and nine different phosphorylated states of CTD sequences with 44 and 14 residues, respectively.

**Figure 1:**
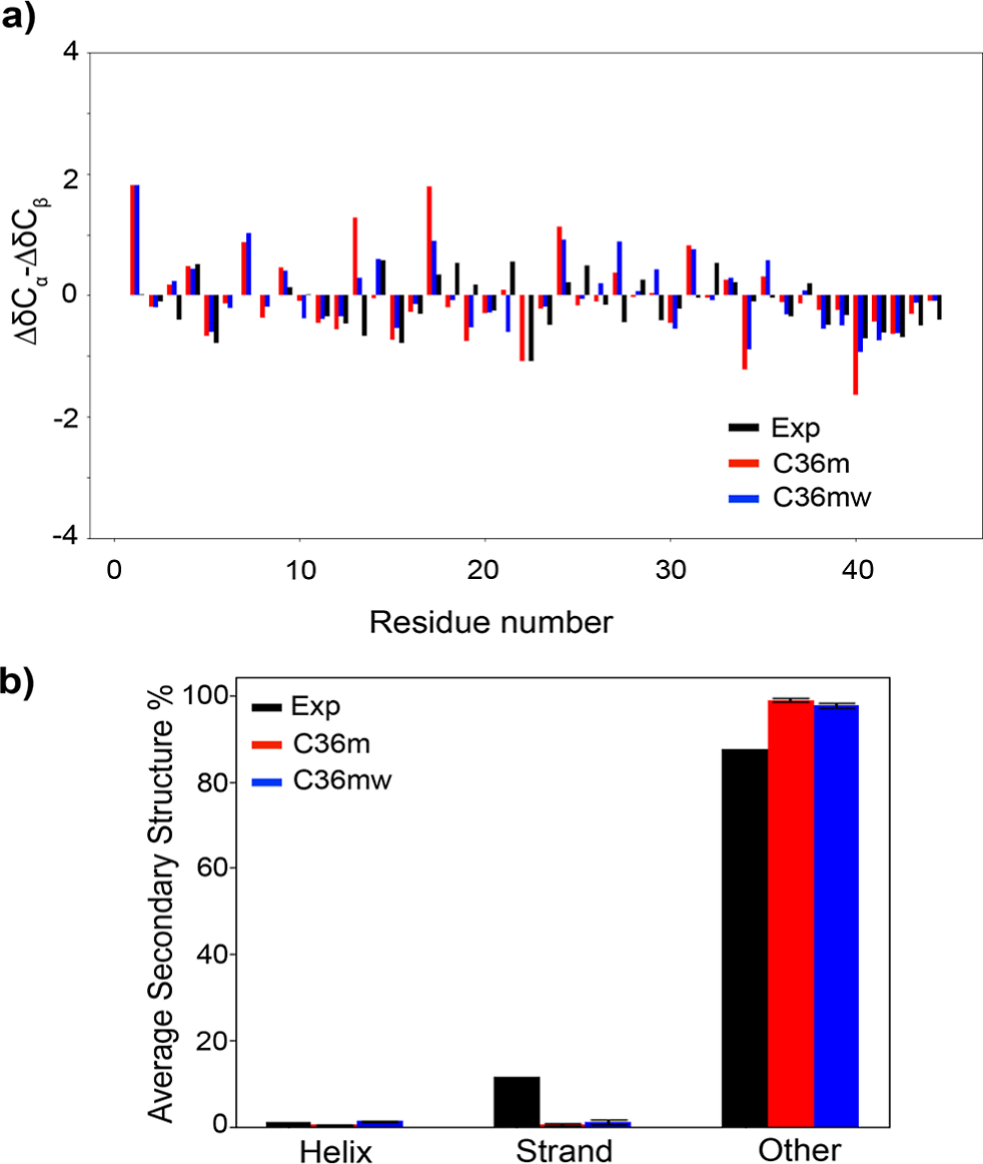
(a) Comparison between the experimental (Exp) secondary chemical shifts^29^ (ΔδC_α_–ΔδC_β_) and the values derived from simulations using both C36m and C36mw FFs as a bar chart representation for exp-CTD-nonphos sequence, and (b) average secondary structure predicted from NMR chemical shifts (experimental with δ2d software) and from simulations using C36m and C36mw FFs with DSSP program (error bars are calculated by splitting the full 200 ns trajectory into 40 ns small trajectories). For DSSP, helix refers to α helix, 3/10 helix, and pi helix, strand refers to isolated beta bridges and extended strands, and other refers to loops, bends, and turns. For the δ2d software, helix refers to α-helix, strand refers to β-strand, and other refers to coil and polyproline II structures.

Each of the CTD sequences in Table 1 was solvated in cubic boxes with a cutoff of 10 Å from each direction of the simulation box to prevent periodic image interactions. Figure S1 shows that both 14- and 44-residue peptides were more than 20 Å from the periodic images throughout the simulations. The systems were neutralized by adding Na^+^ ions when required. The CHARMM-modified TIP3P parameters^43^ were utilized for the explicit water. For the exp-CTD-nonphos system, we used CHARMM C36m^44^ and a modified version^44^ of C36m, we referred it as CHARMM C36mw following an earlier paper that used this modified FF.^45^ C36mw has a modification in nonbonded interactions between protein and water such that the depth potential (ε_H_) is modified from −0.046 to −0.1 kcal/mol for H atoms of water molecules to be applied for water–protein interactions, while the Lennard-Jones parameters for the water oxygen atoms and water–water interactions remain the same with the original CHARMM-modified TIP3P model.^44^ We obtained a similar agreement with the experimental NMR chemical shifts^29^ when using C36m and C36mw FFs (Figures 1 and S2). The main difference is that C36mw provided more extended structures than did C36m (Figure S3). We selected C36mw for the rest of the simulations, mainly because earlier studies reported that c36mw provided better agreement with structural properties of IDPs.^44–46^ Then, an energy minimization was performed for 5000 steps with a 100 kJ/mol tolerance. The systems were equilibrated for at least 625 ps while increasing the temperature from 100 to 300 K. During the equilibration, the backbone and the side chains of CTDs were constrained using a force constant of 400 and 40 kJ/mol/nm^2^, respectively. The simulations were performed using OpenMM^47^ on GPU machines. Long-range electrostatic interactions were calculated using periodic boundary conditions with the particle mesh Ewald (PME) algorithm.^48,49^ The Lennard-Jones interactions were switched between 1.0 and 1.2 nm. The time step was set to 1 fs for the equilibration. The Langevin thermostat was utilized with a friction constant of 1 ps^–1^ in order to maintain the temperature. Table S1 shows the details of the system sizes and numbers of atoms, ions, and water molecules.

### Replica-Exchange Molecular Dynamics Simulations

All of the production simulations were performed using REMD simulations in order to enhance sampling and obtain conformational spaces for CTD models. REMD simulations were performed using the OpenMM^47^ package with GPU-enhanced environments. The final configurations from the previous equilibration step were utilized as the initial configurations for the REMD simulations. For the CTD sequences with 44 residues and 14 residues on Table 1, 16 and 8 replicas were utilized, respectively, in order to maintain the exchange acceptance probability above 30% between replicas (see Table S2). The temperature range for the replicas was set from 300 to 500 K. Langevin dynamics was used as a thermostat with a time step of 2 fs. Long-range electrostatic interactions were calculated using a reaction field approximation^50^ beyond a cutoff distance for the periodic systems. As for the equilibration, the Lennard-Jones interactions were switched between 1.0 and 1.2 nm. At each 500 steps (time intervals of 1 ps), an exchange was attempted during all the REMD simulations. The production REMD runs were performed for 200 ns for each CTD system, except for 2CTD-2P-5P-9P-12P shown in Table 1, which was extended up to 500 ns to obtain a better convergence compared to 200 ns trajectory (Figure S4). For this system, the extended structures were sampled during the first 100 ns of the simulations, as the initial structure was extended. After 100 ns, bend structures became dominantly sampled without any observation of an extended structure for the rest of the simulation (400 ns). This suggests that extended structures are not favorable for this peptide, and therefore, we discarded the first 100 ns of the simulation for this system. To confirm the convergence of other systems, we selected nonphosphorylated 2CTD system as an example and extended the simulations up to 400 ns (Figure S5). We observed that the first and second half of the simulations sampled similar conformational ensembles, further suggesting that convergence of the simulation was achieved within a 200 ns REMD simulation time. Frames were saved every 10 ps during the simulations. Altogether, a total of 34.4 μs of simulations were achieved.

### Data Analysis of the REMD Simulations

The analyses were performed for the trajectories at the lowest temperature (300 K). The radius of gyration (*R*_g_), end-to-end distance, hydrogen bonds (H-bonds) analysis, distance maps, and principal component analysis (PCA) were performed using the MDAnalysis package.^51^ Distance maps were generated using the minimum distances between the residues for each CTD. The PCA was performed using the Cartesian coordinates (backbone atoms) of the CTDs with the first and second principal components (PC1 and PC2). For the PCA, first, the trajectory was prealigned to the initial frame before determining the average structure of the trajectory. Once the average structure was determined, the trajectory was realigned to the average structure. Then PCA was performed using the Cartesian coordinates of the backbone atoms of the protein. The free energy landscapes (using PC1 and PC2 as well as *R*_g_ and end-to-end distance as reaction coordinates) were generated by MATLAB.^52^ The weighted histograms in order to generate free energy landscapes were calculated using the WHAM package developed by the Grossfield lab.^53^ The secondary structures from the simulations were predicted using the DSSP program^54^ in MDTraj.^55^ The secondary structures obtained from the DSSP program were categorized as helix (α helix, 3/10 helix, and pi helix), strand (isolated beta bridges and extended strands), and other/coil (loops, bends, and turns). The experimental secondary structures were predicted using the δ2d software^56^ using available NMR chemical shifts for exp-CTD-nonphos system.^29^ The δ2d software predicted α-helix, β-strand, coil, and polyproline II structures, the latter two of which are referred to as other structures in this work. C_α_ and C_β_ chemical shifts from the simulations were calculated by the SPARTA+ algorithm.^57^ The secondary chemical shifts (ΔδC_α_, ΔδC_β_) were calculated by subtracting the C_α_ and C_β_ chemical shifts for the random coil, which were obtained from the Poulsen Web Server.^58^ Trajectories of the full 200 ns were used for *R*_g_, end-to-end distance, PCA, H-bond calculations, secondary structure predictions, distance maps, and chemical shift calculations except for 2CTD-2P-5P-9P-12P, for which we used the last 400 ns of the full 500 ns trajectory. Central structures were used for comparing secondary structure prediction methods of DSSP, STRIDE,^59^ and KAKSI.^60^ Central structure for each CTD was determined by calculating the average structure of CTD from the trajectory and then calculating the root-mean-squared displacement (RMSD) between the average structure and each frame of the trajectory. Then the frame with a minimum RMSD with respect to the average structure was selected as the central structure. The Na^+^ ion densities around phosphate groups of serine residues were calculated using the VolMap tool in the Visual Molecular Dynamics package (VMD)^61^ (the Na^+^ densities were averaged along the trajectories and mapped as an iso-surface on the central structures of each CTD for clarity). The rotational entropies were calculated from the principal moments of inertia using the CHARMM analysis package.^37^*t* tests were performed using the SciPy module of Python.^62^

## Results

CTD of Pol II is a low complexity domain formed by heptapeptide repeats, as human CTD has 52 repeats, while yeast CTD has 26 repeats. It is computationally challenging to simulate the whole CTD from either human or yeast, while the model CTD with 44 residues (exp-CTD-nonphos), which was experimentally studied, and a CTD with two heptapeptide repeats (2CTD) could potentially provide important insights into the conformations of CTD sequences in general. We performed REMD simulations of the nonphosphorylated and phosphorylated 44-residue CTD and 2CTD models. Below, we first show the agreement of simulation results with the experimental NMR observations for the 44-residue CTD. Then, we present the results from the two CTD models in various phosphorylation states. Finally, we generalize our conclusion by proposing a model to explain the phosphorylation effects on the conformations of CTD models.

### Simulations Predicted Mostly Disordered Conformations Consistent with Experiments

The secondary chemical shifts (ΔδC_α_–ΔδC_β_) calculated from NMR measurements^29^ and our simulations with two FFs (C36m and C36mw) are compared in Figure 1a for exp-CTD-nonphos sequence. Both C36m and C36mw showed good agreement with the experimental C_α_ and C_β_ chemical shifts (Figure S2) and varied agreement with the secondary chemical shifts in Figure 1a, which is similar to the agreements obtained by an AMBER force field reported in the earlier studies.^22,29^ For some residues, both FFs presented a good agreement while there are deviations observed for most of the residues. Overall, secondary chemical shift differences from the experiment and simulations with both FFs are near zero suggesting that the structure is mostly disordered and there is not any significant helical or β sheet propensity.^63^ Moreover, Figure 1b compares the average secondary structure percentages predicted from our simulations (using the DSSP program) and from experimental NMR chemical shifts (using δ2d software). Although secondary structures were predicted using different methods for experiments and simulations, we expect their predictions to be comparable to some extent as both methods were validated against experimental observables.^56,64^ Average secondary structure percentages are in good agreement between experiments and simulations (both C36m and C36mw), specifically with the helix and other structures. However, the average strand structure percentage is higher from the experiments compared to two FFs which is consistent with the previous computational work with AMBER FF.^22^ We note that we used DSSP method for the secondary structure prediction, which is a method developed to predict mostly regular secondary structures, while earlier studies show that DSSP and similar secondary structure prediction methods demonstrated larger disagreements when predicting more disordered structures.^65^ We compared DSSP predictions with other methods, STRIDE^59^ and KAKSI,^60^ in Figures S6 and S7. KAKSI predicted everything in the coil structure, while DSSP and STRIDE provided similar predictions for the disordered regions with some variations in turn and bend. Both FFs provided a mostly disordered conformation that is in good agreement with the experimental results, while it was challenging to discriminate the distinct disordered secondary structures using the available structure prediction methods. Overall, the simulations with both FFs showed a good agreement for C_α_ and C_β_ chemical shifts and secondary chemical shifts as well as provided mostly disordered secondary structures consistent with the experiment, which altogether suggest that both FFs captured the structural features of the CTD model reasonably well.

### Phosphorylation Caused Extended Conformations in CTD Sequences with 44 Residues

**Figure 2:**
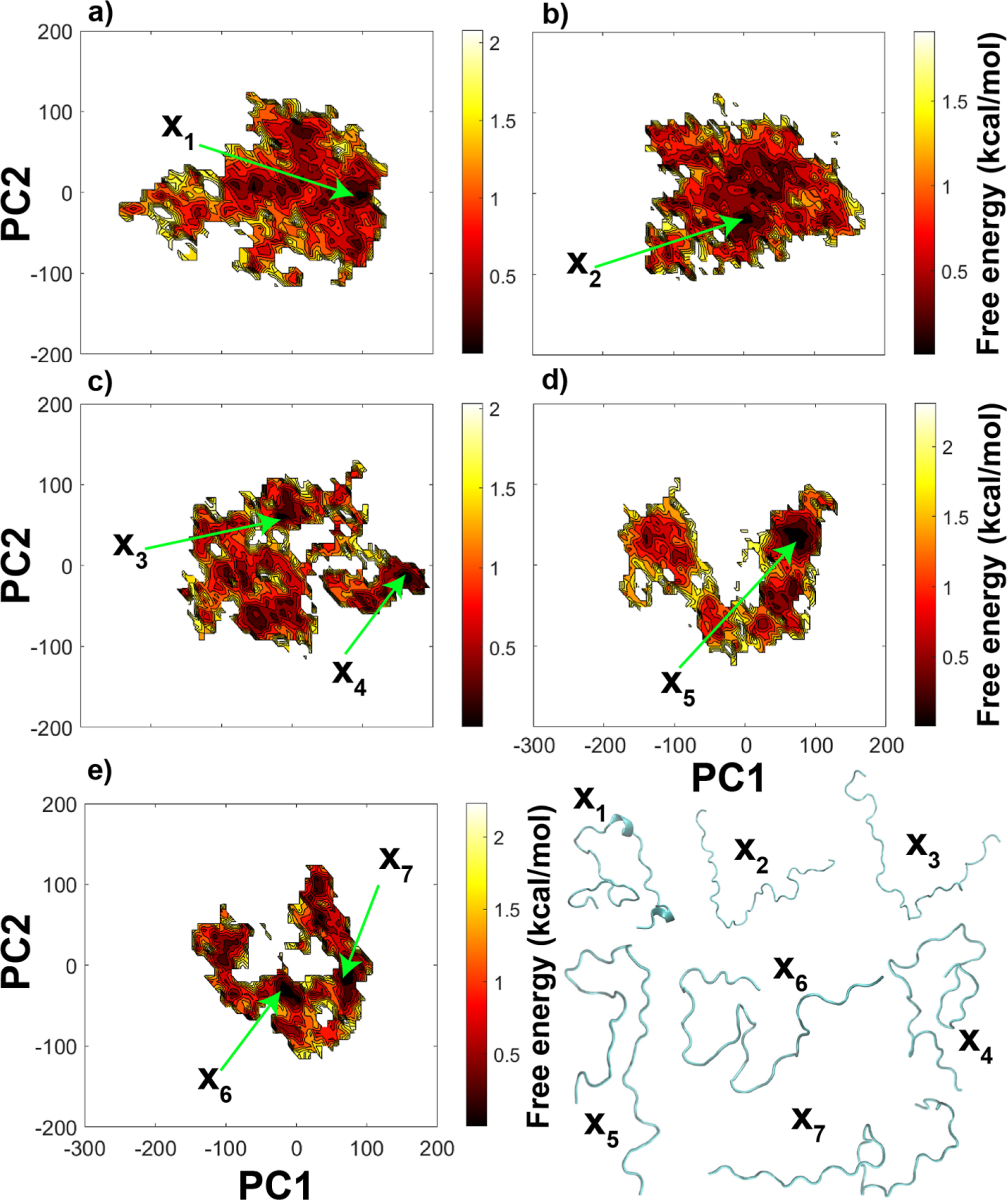
Free energy landscapes using PC1 and PC2 as reaction coordinates from the PCA using Cartesian coordinates of CTDs, (a) exp-CTD-nonphos, (b) exp-CTD-5P-40P, (c) exp-CTD-5P-22P-40P, (d) exp-CTD-5P-12P-18P–32P and (e) exp-CTD-5P-12P-18P-25P-32P-40P. In addition, X_1_–X_7_ represents a few of the lowest energy conformations of different CTD sequences.

We performed REMD simulations of 44-residue CTD (exp-CTD-nonphos) and its four phosphorylated structures (exp-CTD-5P-40P, exp-CTD-5P-22P-40P, exp-CTD-5P-12P-18P-32P, and exp-CTD-5P-12P-18P-25P-32P-40P). In order to obtain the most probable low energy conformations and the conformational space of each CTD sequence, we applied PCA using Cartesian coordinates. PCA is a widely used dimensionality reduction method to represent high-dimensional conformational information into two-dimensional plots to visualize the conformational space that the simulations sampled.^21,23–25,66–68^ PCA of conformations will also provide information about the convergence of the simulations as the energy landscapes should be connected in converged simulations. It is expected to observe a broad free energy landscape from the PCA of IDPs as they span large conformational ensembles compared to structured proteins. Figure 2 shows the free energy landscapes generated by PCA using the first and second principal components (PC1 and PC2 respectively) and the lowest energy conformations in the bottom (X_1_–X_7_). The conformational space for nonphosphorylated CTD represented as a PCA plot in Figure 2a shows a broad landscape, suggesting that CTD has a large conformational ensemble and exchanges conformations without high energy barriers. As the phosphorylation level increases, the conformational landscapes become less broad with multiple distinct minimum energy conformations separated by relatively high energy barriers (Figure 2c–e), suggesting that phosphorylation restricted the conformational space of CTD models. Conformations of phosphorylated CTDs (X_2_ to X_7_) were more extended compared to the nonphosphorylated CTD (X_1_). In addition to this, Figure S8 shows the free energy landscapes for *R*_g_ vs end-to-end distances which suggest that more extended structures were observed upon phosphorylation, regardless of the number and position of the phosphorylation sites.

**Figure 3:**
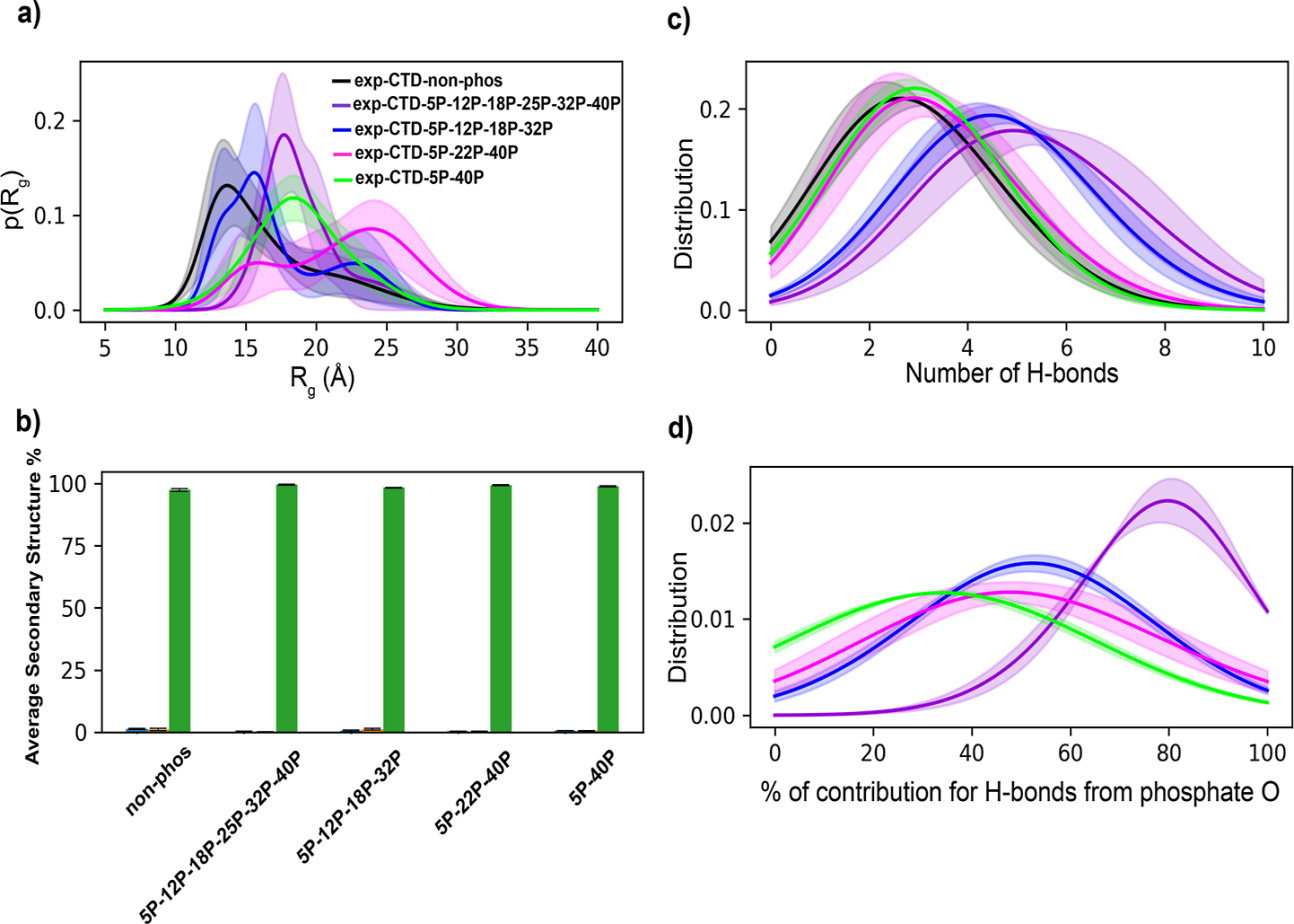
(a) Radius of gyration (*R*_g_) density distributions for CTD sequences with 44 residues, (b) average secondary structure percentages for all the CTD systems with 44 residues as a bar chart representation (blue—helix, orange—strand, and green—coil), (c) distributions of the total number of intrapeptide H-bonds for CTD systems with 44 residues, and (d) distributions of contributions from oxygen atoms of phosphate groups to intrapeptide H-bonds of phosphorylated CTDs with 44 residues. [Colors of the distribution curves are the same as in panel (a) for panels (c,d)]. Standard errors are calculated by splitting the full 200 ns trajectory into 40 ns small trajectories.

Figure 3a, shows the *R*_g_ density distributions with standard errors for CTD sequences with 44 residues. We also provided a comparison of *R*_g_ distributions of each phosphorylation state with the distribution of exp-CTD-nonphos (Figure S9). Additionally, *p*-values from the *t*-test between the *R*_g_ distributions of exp-CTD-nonphos and each phosphorylation state were provided in Table S3. Both error bars and *p*-values suggest that differences in *R*_g_ distributions are statistically significant, except for exp-CTD-5P-12P-18P-32P, in which the differences are within the errors (Figure S9). All the phosphorylated states of the CTD show expansion with respect to the nonphosphorylated state (exp-CTD-nonphos). This observation is expected, as the net charge of exp-CTD-nonphos sequence is negative, and upon phosphorylation, the repulsive interactions between negatively charged residues and the phosphate groups tend to be increased, and consequently the conformations were extended. This observation is also in agreement with the previous computational work done by Jin and Gräter,^69^ that they observed extended structures for the IDP sequences with a negative net charge and around the same length as 44-residue CTD sequences upon phosphorylation. In addition, compared to other phosphorylated states, the exp-CTD-5P-22P-40P sequence shows a broader density distribution of *R*_g_, while exp-CTD-5P-12P-18P-25P-32P-40P has a sharper distribution. This suggests that the relative positions of the phosphorylated residues might play an important role. There is an opportunity to sample more diverse conformations for exp-CTD-5P-22P-40P sequence with well-spread relative positions of the phosphorylated residues. However, an increased number of phosphorylation sites restricted conformational sampling in the case of the exp-CTD-5P-12P-18P-25P-32P-40P model.

Figure 3b represents the overall average secondary structure for the CTDs with 44 residues, and Figure S10 shows the percentages along each residue of the CTD sequence. As expected, all the CTDs are mostly disordered, which can be verified with high percentages (>90%) of coil structures. Here, coil structures include all the loops, bends, and turns according to the DSSP definition.^54^ Also, Figures 3b and S10 show helix structures (α helix, 3/10 helix, and pi helix) and strand structures (isolated beta bridges and extended strands) in lower percentages (<10%). We also generated the secondary structure evolution with time in Figure S11 to understand the stability of these secondary structures of low percentages in Figure 3b (see Figure S10 also). We found that both helix and strand structures remain stable for very short periods of time compared to where in most cases coil structures remain stable for longer periods of time. Moreover, exp-CTD-nonphos shows helix and strand structures for almost all the residues in low percentages and more elevated helix structures around residue IDs 24–32 and 36–44 (see Figures S10a and S11a) compared to the phosphorylated CTDs, which can be also seen in the minimum energy conformation obtained by PCA (see X_1_ conformation in Figure 2). A previous study by Tang et al.^22^ also reported mostly disordered structure with low helix probabilities for the same nonphosphorylated CTD sequence using an AMBER force field. Phosphorylation did not show any significant change in the secondary structures, although there are slightly more elevated strand structures for exp-CTD-5P-12P-18P-32P (Figure S10d) around residue IDs 6–14 and 27–30. Overall, CTDs with or without phosphorylation show high coil structure percentages and low helix and strand percentages; however, there are few specific changes of secondary structure in low percentages upon phosphorylation.

In order to identify the interactions with nearby residues in CTD conformations, we analyzed intramolecular H-bonds. Figure 3c,d show the distributions (with standard errors) of the total number of intrapeptide H-bonds for CTDs with 44 residues and the contributions from the oxygens of phosphate groups to the total number of H-bonds, respectively, and Figure S12 shows the comparison of H-bond distributions of each phosphorylation state with the distribution of exp-CTD-nonphos. We found that the total number of intrapeptide H-bonds formed between residues of CTD increases with the number of phosphorylated residues. Consistently, the contribution to the total number of intrapeptide H-bonds from oxygens of phosphate groups also increases with the number of phosphate groups. In addition to this, Figure S13 shows that the number of close contacts increases around the phosphorylated residues according to the distance maps for exp-CTD-5P-12P-18P-32P and exp-CTD-5P-12P-18P-25P-32P-40P. This shows that the number of overall interactions increases around the phosphorylation sites in the CTDs for highly phosphorylated systems.

### Phosphorylation had Diverse Effects on the Conformation of CTD Sequences with 14 Residues

**Figure 4:**
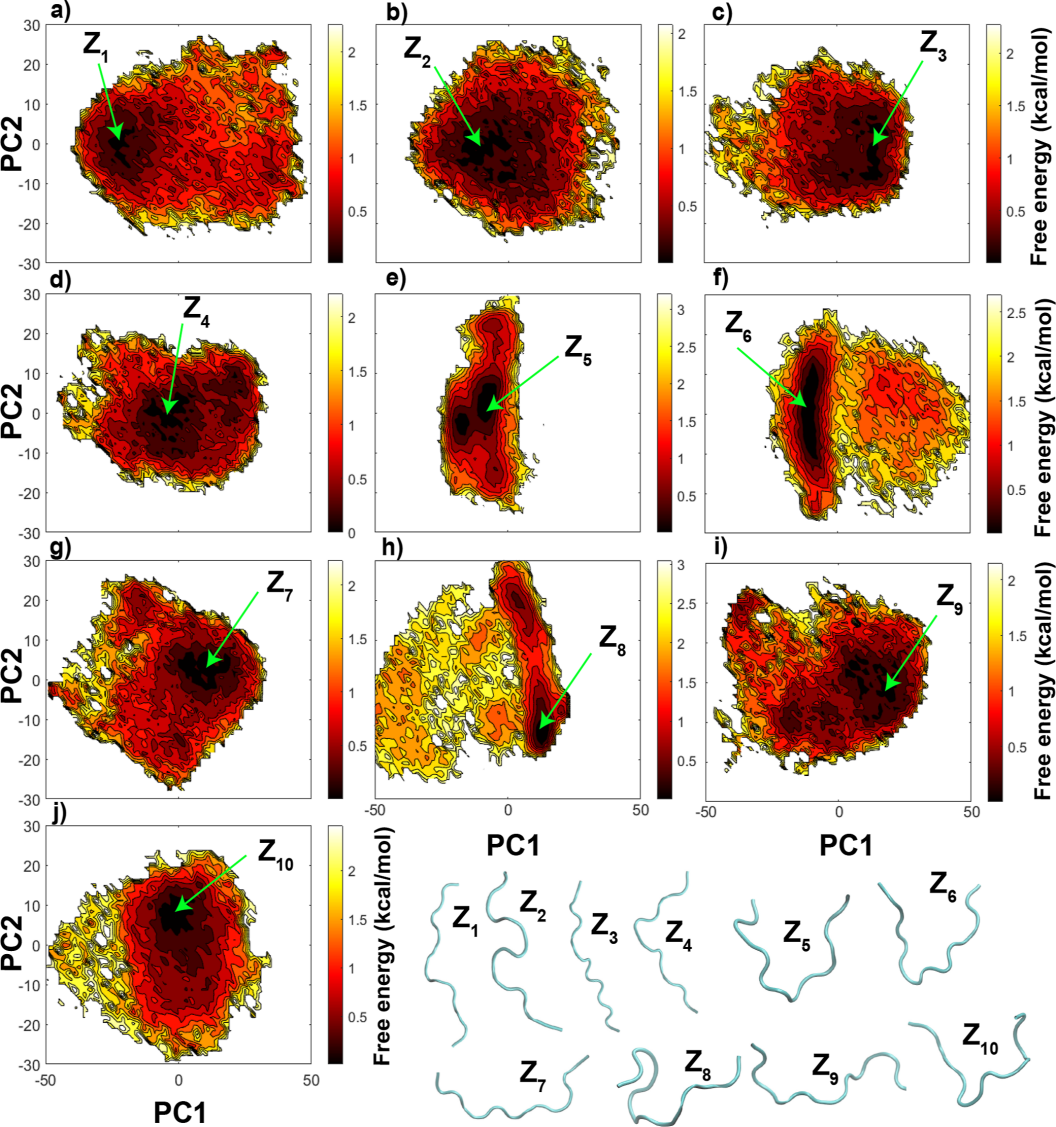
Free energy landscapes using PC1 and PC2 as reaction coordinates from the PCA using Cartesian coordinates of CTDs, (a) 2CTD-nonphos, (b) 2CTD-2P, (c) 2CTD-2P-5P, (d) 2CTD-2P-5P-9P, (e) 2CTD-2P-5P-9P-12P, (f) 2CTD-2P-5P-12P, (g) 2CTD-2P-9P, (h) 2CTD-2P-12P, (i) 2CTD-5P, and (j) 2CTD-5P-12P. In addition, Z_1_–Z_10_ represents a few of the lowest energy conformations of different CTDs.

The CTD model with 44 residues showed a broad conformational landscape and increased extension in the structure upon phosphorylation. In order to cover effects of a larger set of phosphorylation patterns on the conformation, we studied a shorter CTD model, which is 2CTD. Figure 4 represents the free energy landscapes from the principal component analysis and a few of the low-energy conformations for each 2CTD model. All of the free energy landscapes are very broad and mostly exhibit a large conformational space. In addition, local energy minimal regions are also broad and separated by low-energy barriers compared to those of the CTDs with 44 residues. This verifies that 2CTDs have many low-energy conformations that can interchange within each other. The structures in Figure 4 show that 2CTD-2P-5P-9P-12P, 2CTD-2P-5P-12P, 2CTD-2P-12P, and 2CTD-5P-12P have relatively more contracted low-energy conformations that are Z_5_, Z_6_, Z_8_, and Z_10_, respectively (also see A_5_, A_6_, A_8_, and A_10_ conformations in Figure S14).

**Figure 5:**
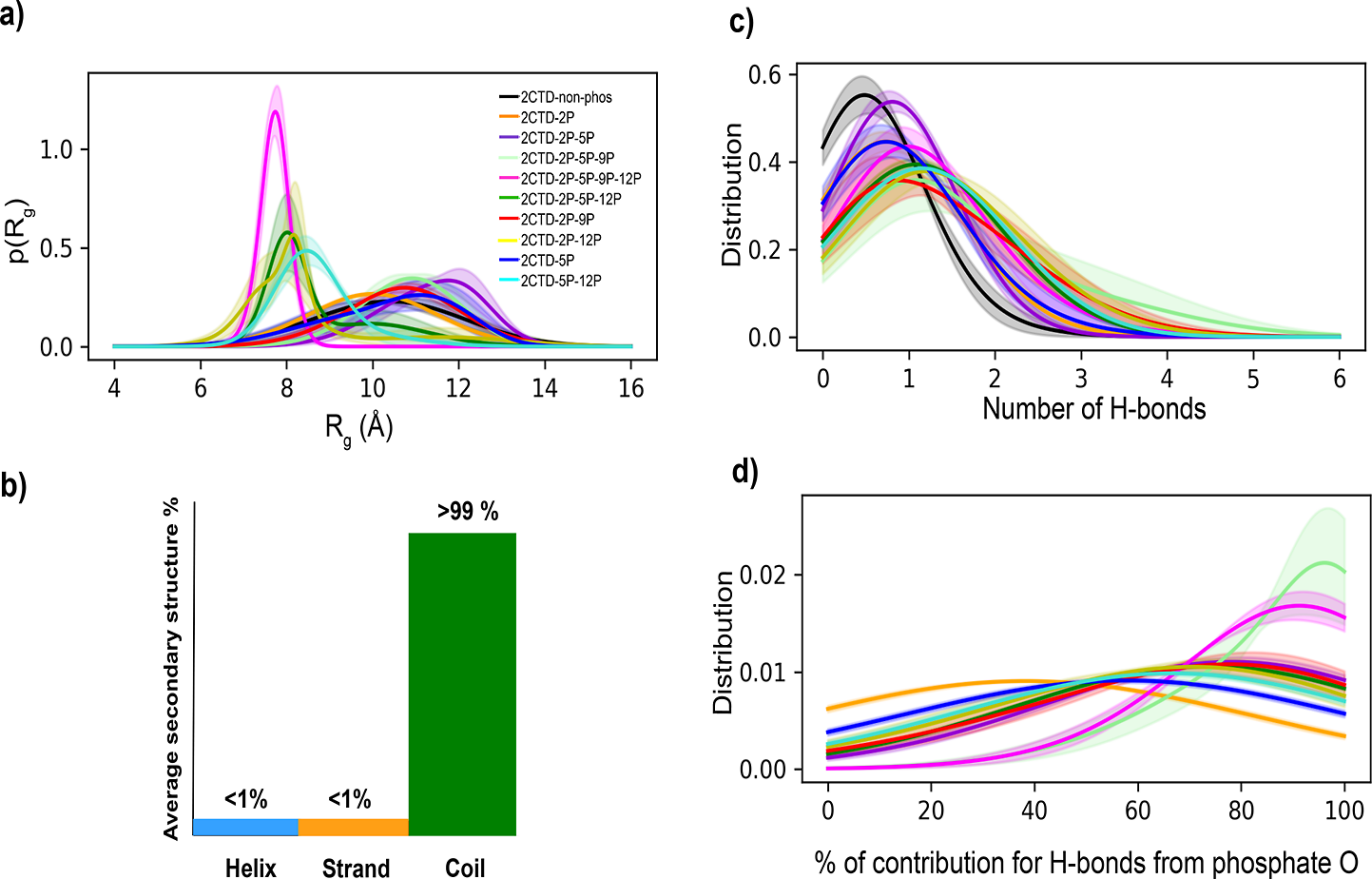
(a) Radius of gyration (*R*_g_) density distributions for CTD sequences with 14 residues, (b) visual representation of average secondary structure percentages for all the 2CTD systems as a common bar chart, (c) distributions of total number of intrapeptide H-bonds for 2CTD systems, and (d) distributions of contributions from oxygen atoms of phosphate groups to intrapeptide H-bonds of phosphorylated 2CTD systems. [Colors of the distribution curves are the same as in panel (a) for panel (c,d)]. Error bars are calculated by splitting the full 200 ns trajectory into 40 ns small trajectories, except for 2CTD-2P-5P-9P-12P, where the 400 ns trajectory was split into 80 ns small trajectories.

Figure 5a demonstrates the density distributions of *R*_g_ for CTDs with 14 residues, and Figure S15 shows the comparison of *R*_g_ distributions of each phosphorylation state with the distribution of 2CTD-nonphos. All the density distributions of *R*_g_ are very broad for CTDs with 14 residues, except for 2CTD-2P-5P-9P-12P, suggesting that the conformations for most of the 2CTDs are interconverting within a large conformational space. Phosphorylation in the shortened CTD results in complex changes in *R*_g_. 2CTD-2P-5P, 2CTD-2P-5P-9P, and 2CTD-2P-9P show expansion upon phosphorylation, while a few of the 2CTDs show contraction, mainly the sequences 2CTD-2P-5P-9P-12P, 2CTD-2P-5P-12P, 2CTD-2P-12P, and 2CTD-5P-12P. These observations suggest that for the CTD with a shortened length (compared to 44 residues), in addition to the net charge and the number of phosphorylated residues, the relative positions of phosphorylation sites also determine whether the peptide will expand or contract. Interaction with nearby residues and ion coordination might be some of the factors that are contributing to the changes in conformational space upon phosphorylation. A similar type of observation was discussed in a previous computational study by Rieloff and Skepö for IDPs around similar lengths.^23^ In that study, they observed an expansion for Tau1 (IDP with positively charged nonphosphorylated state and 11 residues) and a contraction for β-casein (IDP with negatively charged nonphosphorylated state and 25 residues) after phosphorylation.

Figure 5b is a visual illustration showing that the average secondary structures are predicted to be over 99% coil for each peptide. Figure S16 shows that for all the 2CTDs, the average coil structure (loops, bends, and turns) percentage is more than 99%, and the average helix (α helix, 3/10 helix, and pi helix) and strand (isolated beta bridge and extended strand) percentages are less than 1% for every residue, which suggests that all the 2CTDs are more disordered than the CTDs with 44 residues (>90% average coil structure). Even for a few of the 2CTDs, the average coil structure percentage is 100% (see Figure S16c–e). Also, the helix and strand structures predicted for 2CTDs are only stable for very short periods of time, as shown in Figure S17.

In order to understand the interactions behind the conformational changes in 2CTD upon phosphorylation, we analyzed intrapeptide H-bonds (Figures 5c and S18). Figure 5c shows that there is an increase in the total number of intrapeptide H-bonds upon phosphorylation of 2CTDs, as we observed for CTDs with 44 residues. In some cases, the increase of H-bonds is not significant (2CTD-2P, 2CTD-2P-5P, and 2CTD-5P in Table S4), while, in other cases, the increase was more than 2-fold (see 2CTD-2P-5P-9P, 2CTD-2P-5P-9P-12P, 2CTD-2P-5P-12P, 2CTD-2P-9P, 2CTD-2P-12P, and 2CTD-5P-12P in Table S4). We observed a similar pattern of increments in CTDs with 44 residues in Table S4. This means in some cases, the phosphorylation significantly induces the number of intrapeptide H-bonds formed in CTDs with both 44 and 14 residues, which will eventually determine their conformations. Also, as we observed for CTDs with 44 residues, the contribution to the total number of intrapeptide H-bonds is mostly from the oxygens of phosphate groups for 2CTD systems (see Figure 5d). Distance maps in Figure S19 show that there are few close contacts that appeared and are specifically linked with the phosphorylated residues for 2CTD-2P-5P-9P-12P and 2CTD-5P-12P which are the two 2CTD models with contractions compared to 2CTD-nonphos in Figure 5a.

**Figure 6:**
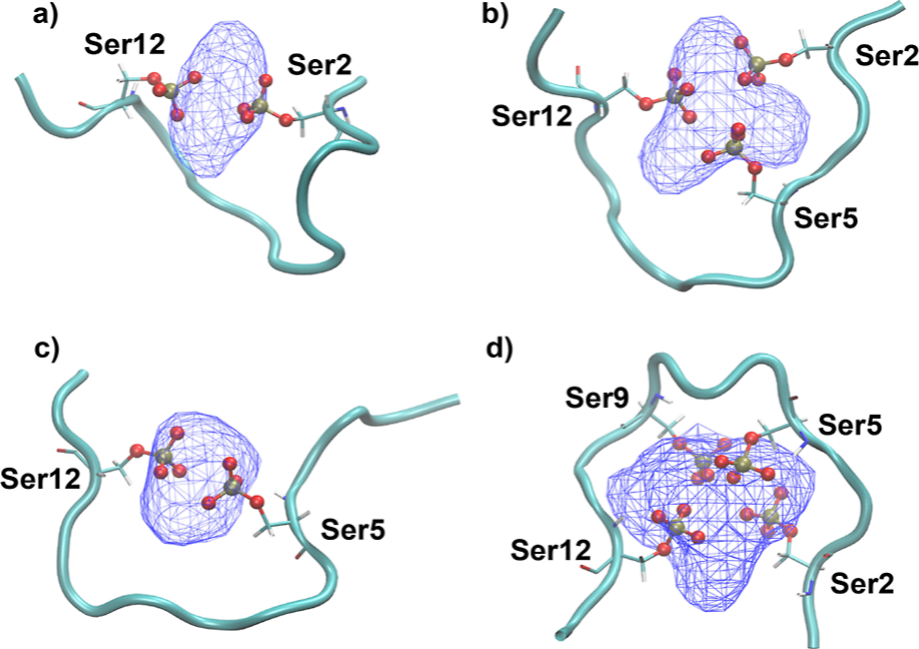
Average Na^+^ ion density around the phosphate groups in serine (Ser) residues for the central structures of contracted 2CTDs. (a) 2CTD-2P-12P, (b) 2CTD-2P-5P-12P, (c) 2CTD-5P-12P, and (d) 2CTD-2P-5P-9P-12P. Blue wire mesh represents the density of Na^+^ ions around the phosphate groups of the highlighted serine residues of each 2CTD sequence. The cyan cartoon structure shows the backbone of each 2CTD sequence. Red and yellow spheres represent oxygen and phosphorus atoms, respectively. The visualizations were generated using the visual molecular dynamics (VMD) package.^61^

In order to understand the electrostatic interactions that potentially stabilize the contracted conformations upon phosphorylation, we analyzed the Na^+^ ion densities. Figure 6 shows the average Na^+^ ion density around phosphate groups of the central structures of contracted 2CTDs, which are 2CTD-2P-12P, 2CTD-2P-5P-12P, 2CTD-5P-12P, and 2CTD-2P-5P-9P-12P. This figure demonstrates Na^+^ ion coordination by the negatively charged phosphorylated Ser residues and bending of the structure as a result of this coordination. This result provides an explanation for the decreased *R*_g_ values and increased contractions of the conformations observed in these specific 2CTD phosphorylated models shown in Figure 5a. As we visualize the low-energy conformations specifically for 2CTD-2P-5P-9P-12P, 2CTD-5P-12P, 2CTD-2P-12P, and 2CTD-2P-5P-12P, interactions with Na^+^ ion makes the phosphorylated residues come closer to each other, which would be energetically unfavorable otherwise due to the presence of repulsive forces as the phosphate groups are negatively charged. Compared to the 44-residue CTDs, due to the shortened length of 2CTDs, Na^+^ ions can form stable complexes with phosphate groups through electrostatic interactions, when phosphate groups are especially present near the two terminal ends of 2CTDs. All the panels in Figure 6 show high Na^+^ ion density around phosphate groups, which eventually induce more compact conformations for the above-mentioned 2CTDs. In contrast, the other 2CTD models show more widely distributed Na^+^ ion densities that do not support any bending and contraction in the structures (see Figure S20). The bottom line is that the shortened length of 2CTDs, combined with the spacings between multiple phosphorylated sites, allows some of the 2CTDs to have more contracted conformations with the help of the formation of Na^+^-phosphate group complexes compared to the nonphosphorylated state, even though the net charges of those phosphorylated 2CTDs are negative.

### Length of CTD and Relative Positions of Phosphorylation Sites Affect the Conformations

**Figure 7:**
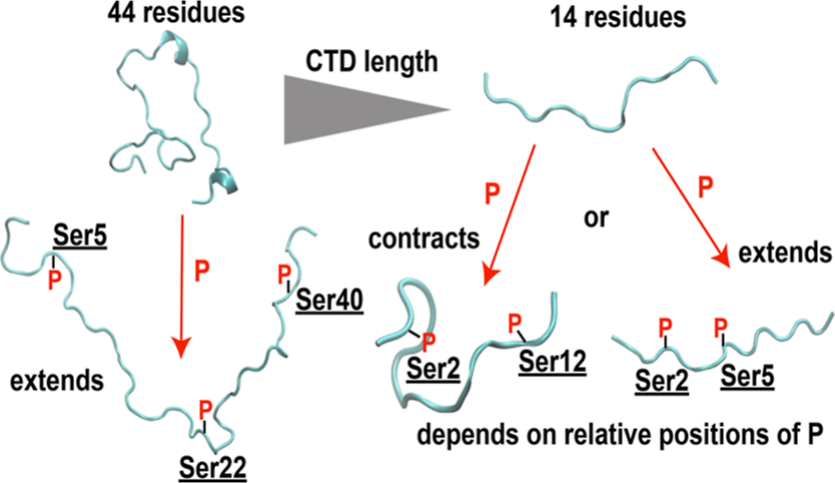
Model for the effects of phosphorylation in conformations of CTDs at different lengths and phosphorylation patterns.

We observed that phosphorylation of CTD with 44 residues caused extended structures, while CTD with 14 residues had either extended or contracted structures upon phosphorylation. A simple model shown in Figure 7 suggests an explanation of the distinct effects of phosphorylation. As the ion density analysis showed, the 2CTD structures bend when multiple phosphorylation sites are in a certain distance range (7–10 residues apart) to coordinate Na^+^ ions by negatively charged oxygen atoms. Bending of the structures causes more compact conformations for such cases. In contrast, for the 44-residue CTDs, phosphorylated structures tend to be extended, and bending is not supported, potentially due to the high entropic cost to bend longer disordered structures. To quantify the entropic cost for bending of the 2CTD systems, we calculated the rotational entropies throughout the simulations. Figure S21 shows that the entropies decrease for the systems in which bending was observed compared to the 2CTD-nonphos system. This supports that the bending of the structures will have an entropic cost and we hypothesize that this entropic cost could be higher in longer chains. Although we observed local bending for closely located phosphorylated residues for the 44-residue CTD (Figure S22), the structures were extended upon phosphorylation. We note that phosphorylation densities of the 44-residue CTD are at the lower end compared with the 2CTD systems (Table S5). We found that extension of the 44-residue CTD somewhat decreases as the phosphorylation densities increase, suggesting that counterion condensation and consequently bending of the close-by residues (Figure S22) may reduce the extension of the structures with high levels of phosphorylation. We also note that, for the 2CTDs, not only the distance between the phosphorylation sites, but also their relative locations affect the compactness of the structure. For example, the effects of phosphorylation for 2CTD-5P-12P and 2CTD-2P-9P are different, although their phosphorylation sites are both 7 residues apart (Figures 6 and S20). Ion coordination by two phosphorylated serine residues of 2CTD-5P-12P supported bending, while in the 2CTD-2P-9P system, the phosphorylated serine residues at positions 2 and 9 were coordinating Na^+^ ions separately (Figure S20). In this case, the hydrogen bonds between phosphorus oxygens at position 9 and the neutral serine residue at position 7 stabilize the structure and prevent bending. Overall, our model suggests that ion coordination can cause contraction in the short CTD structures, whereas for the longer structures, contraction was restricted due to the combination of electrostatic repulsion and the entropic cost for bending.

## Discussion

In this study, we generated conformational ensembles of CTD models at two different lengths and varying phosphorylation states by using enhanced sampling MD simulations. REMD simulations at all-atom details provided a large span of the conformational ensemble for both 14- and 44-residue CTD models. As secondary structure predictions of 44-residue CTD show (Figure 1), the five blocks of simulations provided small standard errors that suggest the convergence of the simulations. We also obtained small standard errors for *R*_g_ and H-bond distributions for both 14- and 44-residue CTDs, that further support the convergence of the simulations. Additionally, secondary structures evolve through time (Figures S11 and S17) suggesting that coil structures are predominant while helix and beta structures are formed transiently, which also support the convergence of secondary structures for the simulations. Also, an extension of the simulation for the nonphosphorylated 2CTD model shows that twice-long simulations provided similar conformation landscapes, further suggesting the convergence of the simulations (Figure S5). However, the computational cost for CTD models substantially increased for the 44-residue CTD as we needed to run 16 replicates to cover the most probable conformational spaces. Therefore, generating the full span of conformational space for the yeast or human CTDs, which have 26 and 52 heptapeptide repeats respectively, is computationally challenging using atomistic REMD simulations. Alternative strategies can be applied for studying full-length CTDs, which include coarse-grained MD simulations,^16,70–72^ fragmentation of long chains,^13,14^ or generative machine learning models^19–21^ using variational autoencoder or attention-based approaches. A future direction for our study is to generate conformations of longer CTD models with different phosphorylation patterns using coarse-grained simulations and then develop a machine learning model based on coarse-grained conformations to predict conformational ensembles of CTD sequences of any given length and phosphorylation pattern. Similar studies showed that such approaches successfully predict the conformations,^19–21^ while none of these studies include post-translational modifications.

CTD of Pol II is known to undergo several post-translational modifications, including phosphorylation of serine residues at the second, fifth, and seventh positions of the heptapeptide repeat. There is a large number of possible combinations of phosphorylation within full-length CTDs, and it is not entirely known which phosphorylation patterns can take place together or which ones are mutually exclusive in vivo.^26,73^ In our study, we selected all the possible 2Ser and 5Ser combinations from the N- to C-terminal ends for 2CTD model as the 2Ser and 5Ser positions are known to be the most observed phosphorylation sites for CTD.^74,75^ However, we note that some of the phosphorylation states we explored in this study may not take place in vivo and, therefore, may be biologically irrelevant. Although the CTD phosphorylation pattern is not entirely clear, some studies suggest that monophosphorylation is more common, and adjacent phosphorylation increases the prevalence of double phosphorylation in a repeat;^74^ and phosphorylation levels are distributed evenly across the sequence that similar amount of phosphorylation is observed close to the Pol II core and at the end of the tail.^75^ Phosphorylation pattern may also be related to the steric hindrance or accessibility of the positions for the kinases, which are the proteins that catalyze phosphorylation. Regardless of the biological feasibility of the phosphorylation patterns, we explored a large set of potential positions for the 2CTD to obtain some generalized rules for the effects of phosphorylation on the conformations.

We proposed a model to describe the effects of phosphorylation on the conformations of CTDs at different lengths. Phosphorylation introduces an increased electrostatic repulsion, which causes an extension of the structure in a longer CTD (44-residue) as expected, while the effects are more complicated for a relatively shorter CTD (14-residues) as the repulsive interactions can be compensated by Na^+^ ion coordination. We concluded that the contraction of the structures is allowed in the 2CTDs due to the counterion condensation but not in the 44-residue CTDs as the entropic cost for bending is expected to be relatively smaller for 2CTD. However, if we go to even longer sequences, the conformational space will be larger, and there could be more complicated alterations in conformations at different phosphorylation patterns. But, overall, we expect that there would be even higher entropic barriers for a significant bending for the longer CTDs upon phosphorylation, and consequently, more extended structures can form, as was reported earlier.^32^ The counterion effects on the conformations were widely studied for highly charged biomolecules, especially for nucleic acids.^76,77^ Studies on IDPs showed that the presence of ions affects the conformations by either ion condensation that reduces the effective charges^78^ or electrostatic screening that reduces the salt bridges formed by oppositely charged residues.^23,24^ We performed the simulations at neutral conditions, that only counterions are present in the systems, and observed a counterion condensation over the negatively charged phosphate groups, which reduced the electrostatic repulsion and caused compaction of the peptides. Compaction of the IDPs upon phosphorylation was observed by previous studies but through salt–bridge interactions with the positively charged residues rather than ion-condensation.^23,24^ At higher salt concentrations, we would expect altered conformations in both long and short CTDs toward more random coil structures due to the screening of the electrostatic interactions. Additionally, our model suggests that charge patterning is crucial in determining the conformation of the CTD models, as suggested earlier.^79^ However, we showed that conformations depend on not only the phosphorylation pattern but also sequence specificity, as surrounding residues also have an impact in addition to the charge effect of the phosphate groups. For example, we observed that phosphates within seven residues apart either collectively coordinate Na^+^ ion by bending (2CTD-5P-12P) or they are stabilized by H-bond interactions with the nearby residues and do not bend (2CTD-2P-9P). Furthermore, our model does not include other factors that can affect conformations, including the binding of CTD to other proteins and the crowding of the cellular environment. CTD of Pol II is known for interacting with other proteins, such as the mediator complex, capping enzymes, transcription, and elongation factors.^26,80^ The conformation of the CTD may be altered upon binding, which is not addressed by our model. As a last point, highly concentrated cellular systems may also induce relatively more compact structures for CTDs and may force intermolecular CTD interactions that may alter the conformations as well.

CTD of Pol II is also well recognized for its involvement in LLPS formation^27,28^ and phosphorylation of CTD was suggested to regulate such phase separation events.^28,81^ Therefore, it is crucial to determine conformational changes upon phosphorylation to better understand its effects on phase separation. To investigate phase separation by CTD, coarse-grained models in conjunction with enhanced simulation techniques can be applied, as was done extensively in recent studies for similar systems.^82–84^ However, available coarse-grained models may need to be fine-tuned for CTD sequences and especially for phosphorylated serine residues. One way to do this is to parametrize a coarse-grained model against all-atom MD simulations of concentrated CTD systems, which will be one of our future research directions.

## Conclusions

We report computationally generated conformational ensembles of Pol II CTD at different lengths and phosphorylation states. We predicted highly disordered structures for all the systems, with predictions of less than 10% of helix and beta strand structures. Introduction of phosphate groups on the serine residues caused more extended structures for the long CTD, while contraction of the structure is observed for some of the short CTD systems. We proposed a model that summarizes the effects of phosphorylation on the conformation of CTD systems. According to our model, Na^+^ ion coordination by multiple phosphate groups takes place depending on the relative positions of the phosphorylation sites, and it stabilizes bending structures for short CTDs, that causes contraction. On the other hand, long CTD extends its structure upon phosphorylation, potentially due to the increased electrostatic repulsion and entropic cost for bending. Future studies will focus on simulating CTD models in concentrated systems to fine-tune coarse-grained models, which will be later used to obtain conformations of full-length CTDs and study LLPS formation by CTD interactions.

## Supporting Information Available

Minimum distance between periodic images along the simulations for the most extended sequences; linear regression analysis for C_α_ and C_β_ chemical shifts from experiments and simulations; comparison of free energy landscapes using *R*_g_ and end-to-end distance as reaction coordinates between C36m and C36mw FFs for exp-CTD-nonphos; comparison of PCA profiles for 2CTD-2P-5P-9P-12P from 100, 400, and 500 ns trajectories; comparison of PCA profiles for 2CTD-nonphos from 200 and 400 ns trajectories; bar chart and visual comparison of the secondary structure predictions using DSSP, STRIDE, and KAKSI; free energy landscapes using *R*_g_ and end-to-end distance as reaction coordinates for CTDs with 44 and 14 residues; distribution of *R*_g_ and H-bonds with standard errors for CTDs with 44 and 14 residues; secondary structure time evolutions and average secondary structure % along sequences for CTDs with 44 and 14 residues; distance maps generated using the minimum distances between the residues for CTDs with 44 and 14 residues; average Na^+^ densities around phosphate groups of other 2CTDs compared to Figure 6 in main text and phosphate groups of CTDs with 44 residues; distribution of rotational entropies for 2CTDs; table of system size details for each simulation; table of total acceptance ratios for CTDs with 44 and 14 residues for REMD simulations; table of *p*-values of *t* tests for CTDs with 44 and 14 residues; table of average number of intrapeptide H-bonds for CTDs with 44 and 14 residues; and table of phosphorylation densities for CTDs with 44 and 14 residues (PDF)

## Notes

The initial structures for nonphosphorylated systems, along with the scripts to generate them, an example of REMD simulation scripts, analysis scripts, and most probable conformations obtained from the PCA analysis in PDB format, were provided in a GitHub repository (https://github.com/bercemd/ctd_conformations).
