# Supplementary material for: Complex Conformational Space of the RNA Polymerase II C-Terminal Domain upon Phosphorylation
Source: J Phys Chem B. 2023 Oct 23;127(43):9223–35. doi: 10.1021/acs.jpcb.3c02655 (PMC10626582; doi:10.1021/acs.jpcb.3c02655)
Supplement: Supplementary file 1 — jp3c02655_si_001.pdf [file jp3c02655_si_001.pdf]

Supporting Information for: Complex Conformational Space of RNA Polymerase II C-terminal Domain upon Phosphorylation

Weththasinghage D. Amith[a] and Bercem Dutagaci[a*]

[a]Department of Molecular and Cell Biology, University of California, Merced, Merced, CA 95343, USA.

[*] Corresponding author:
Bercem Dutagaci
5200 North Lake Rd.
Merced, CA 95343, USA
bdutagaci@ucmerced.edu
209-228-3603

a)

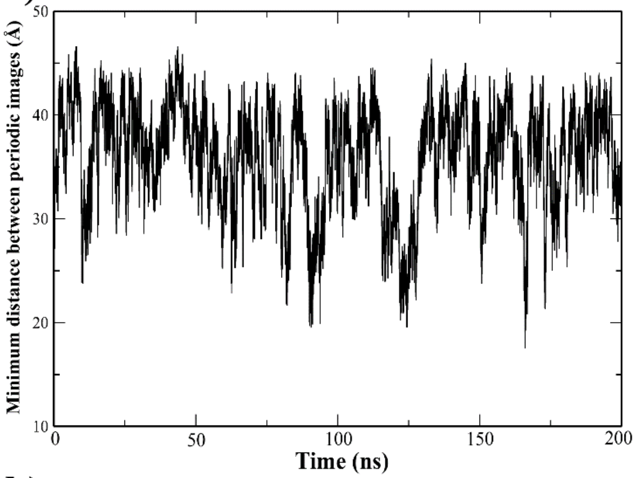

b)

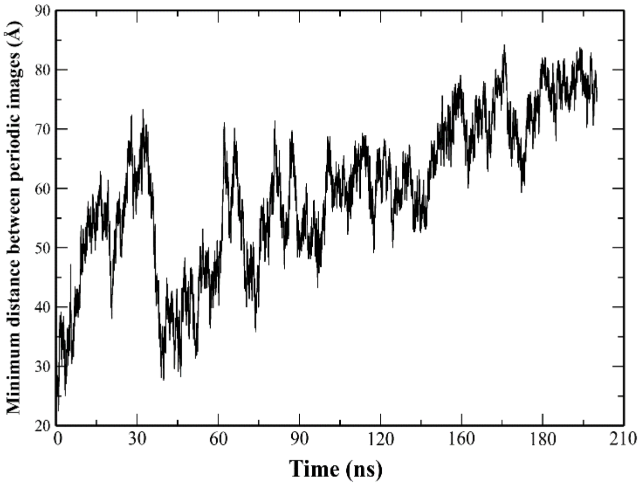

Figure S1. The minimum distance between periodic images along the simulation time for the most expanded sequence from 2CTDs and exp-CTDs. a) 2CTD-2P-5P and b) exp-CTD-5P-22P-40P.

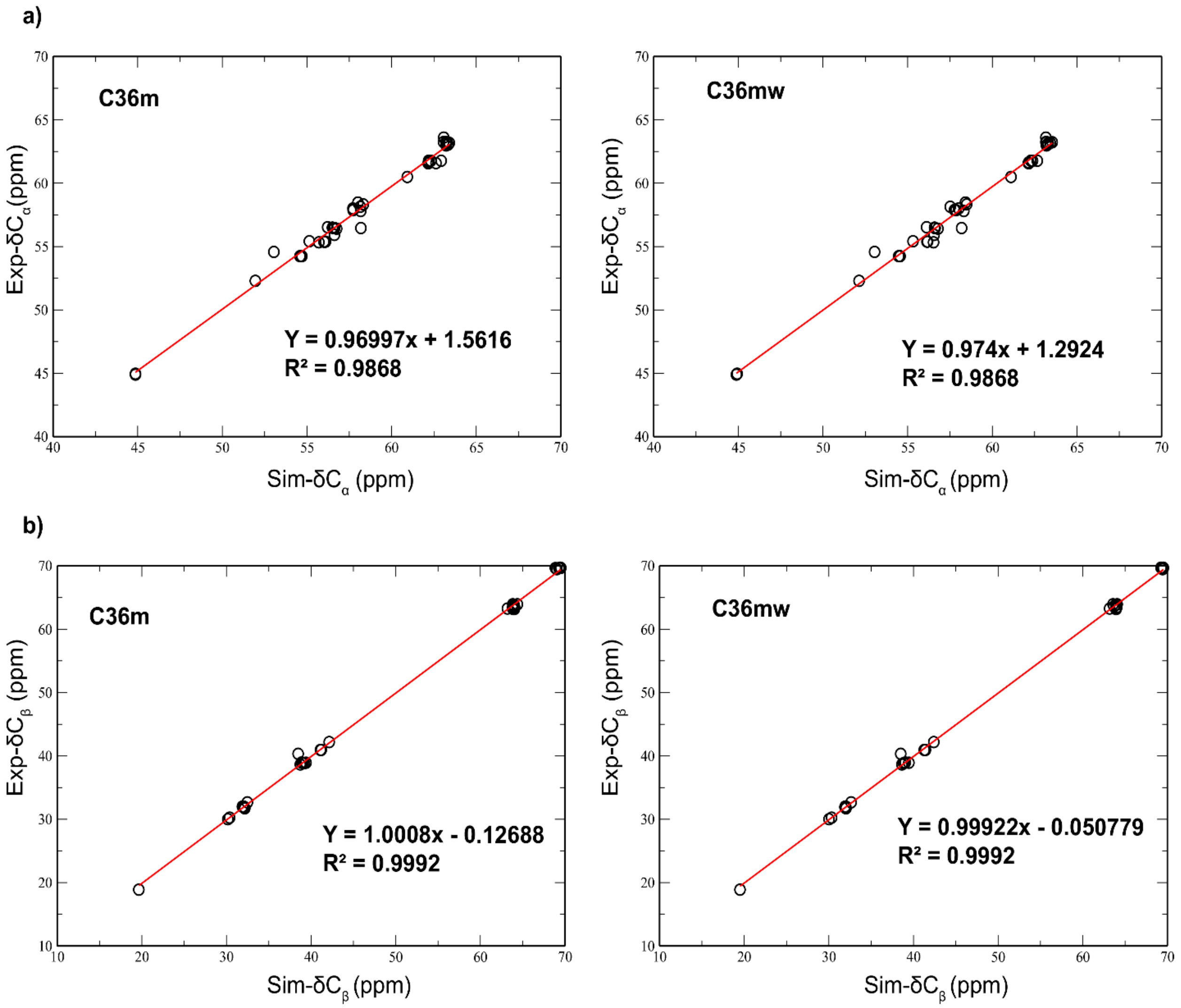

Figure S2: Comparison of the correlations of chemical shifts of a) C_α and b) C_β between experimental values and chemical shifts determined from C36m and C36mw FFs for exp-CTD-non-phos sequence with linear regression analysis (trendlines are shown using red solid lines and the equations for the trendlines and R^2 values are displayed on the plots).

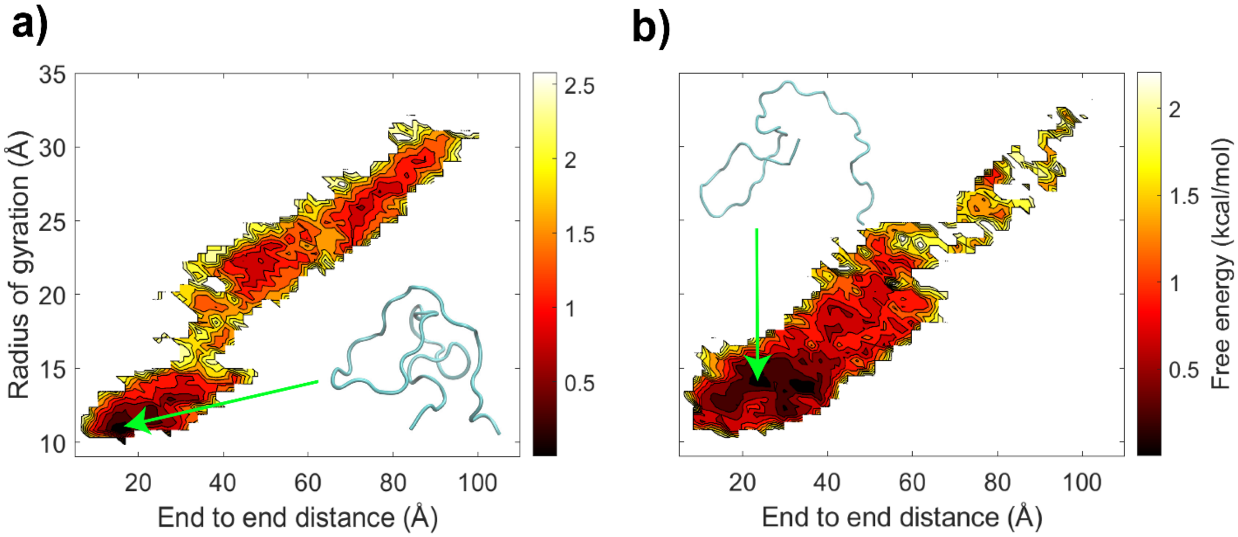

Figure S3: The free energy landscape using radius of gyration and end to end distance as reaction coordinates for exp-CTD-non-phos with 44 residues from the simulations with C36m (a) and C36mw (b) force fields. Low energy conformations are shown in cartoon representations.

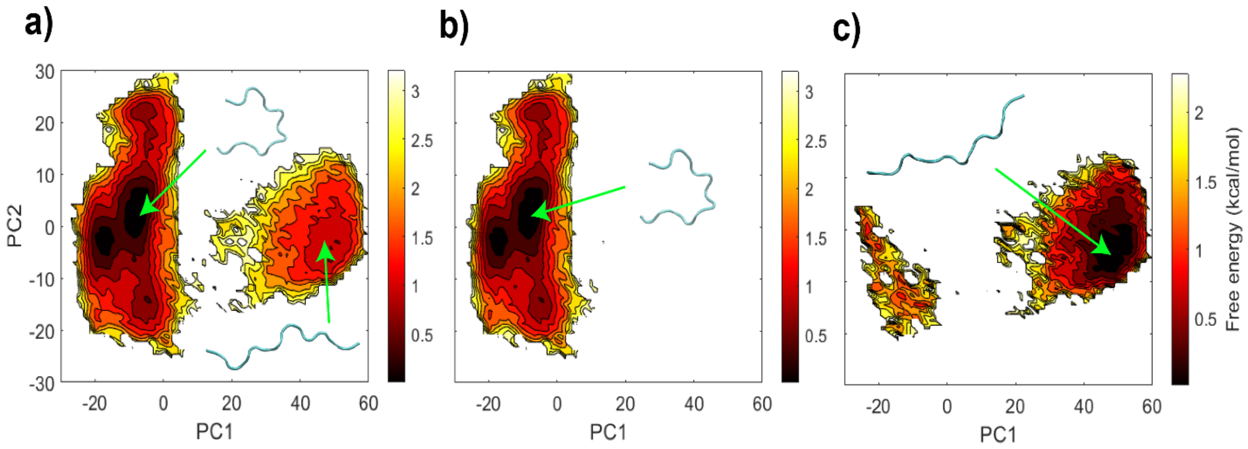

Figure S4: The free energy landscapes from PCA analysis for 2CTD-2P-5P-9P-12P system using (a) full 500 ns of the simulation, (b) last 400 ns and (c) first 100 ns of the 500 ns simulation. The low energy conformations are also shown in each panel with cartoon representation.

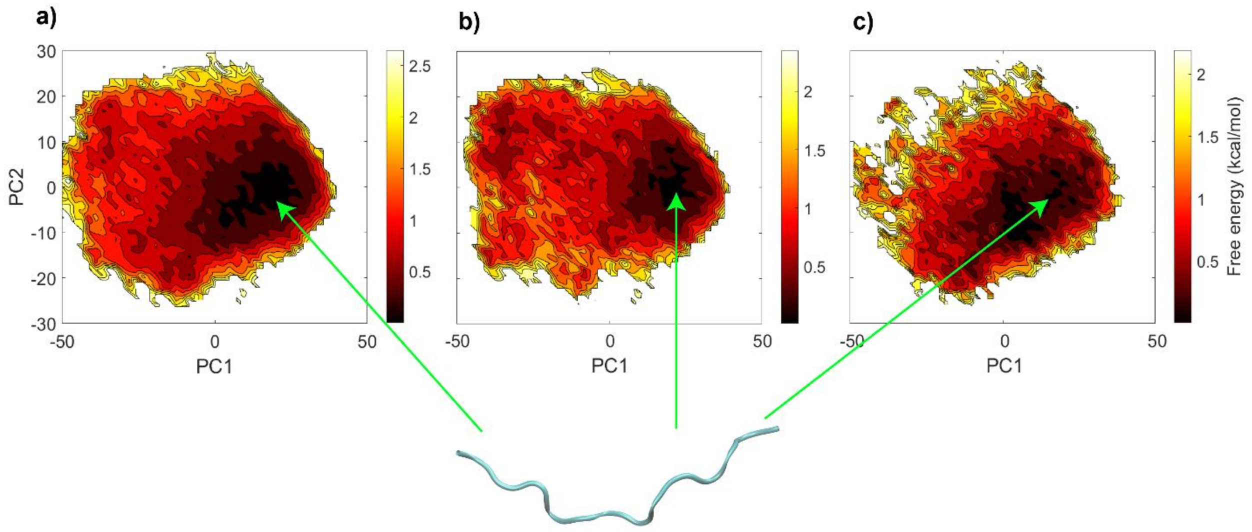

Figure S5: The free energy landscapes from PCA analysis for 2CTD-non-phos system using extended 400 ns of the simulation; (a) full 400 ns, (b) first 200 ns and c) last 200 ns of the 400 ns simulation. One of common low energy conformations is also shown with cartoon representation.

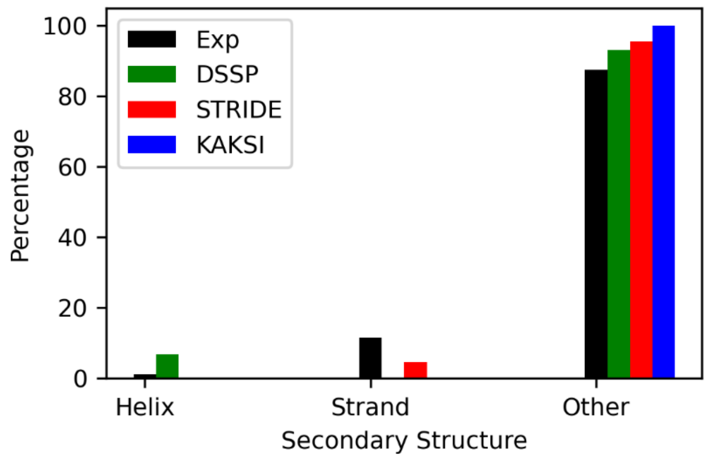

Figure S6: Comparison of the secondary structure predictions from NMR chemical shifts (experimental with δ2d software) and for the central structure from the simulations using C36mw FF with the DSSP, STRIDE and KAKSI programs.

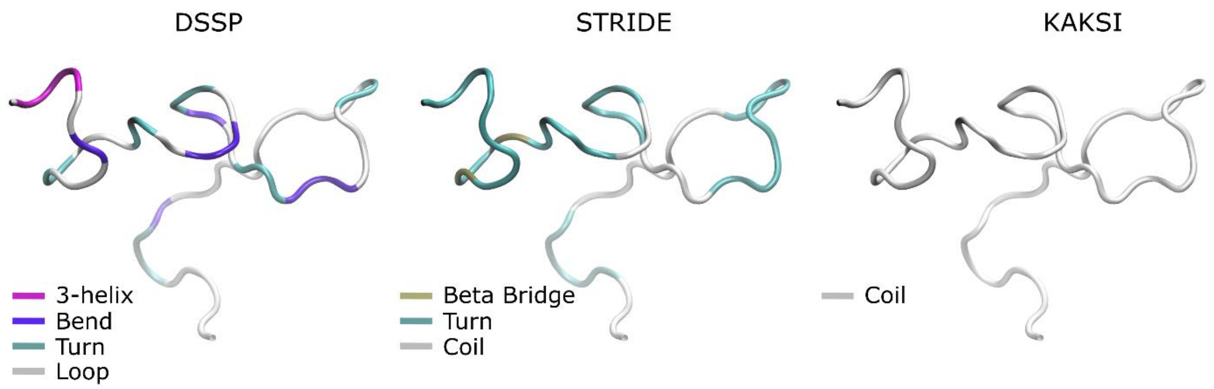

Figure S7: Visual comparison of the secondary structure predictions for the central structure from the simulations using C36mw FF with DSSP, STRIDE and KAKSI programs.

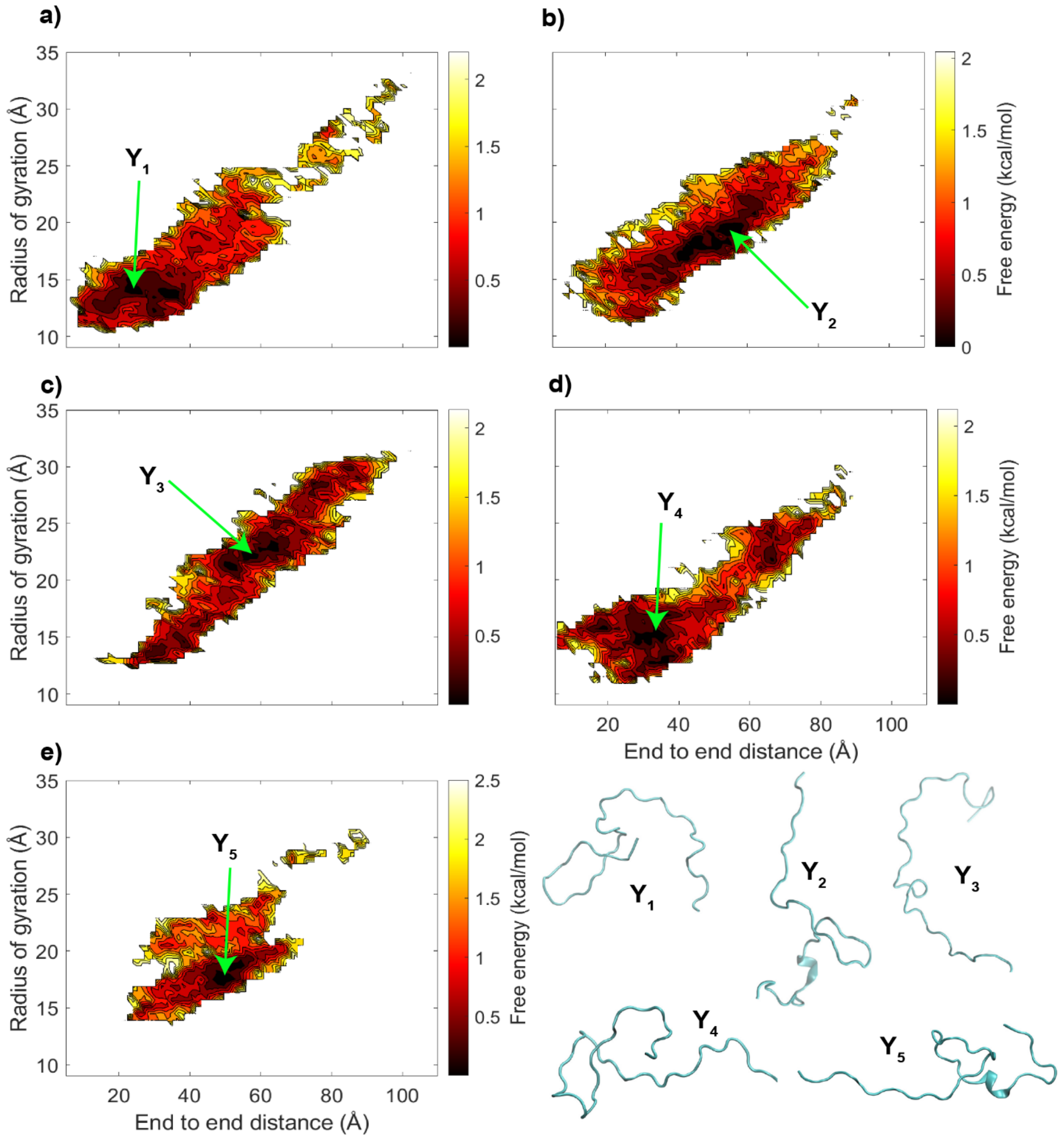

Figure S8: The free energy landscapes using radius of gyration (R_g) and end to end distance as reaction coordinates, a) exp-CTD-non-phos, b) exp-CTD-5P-40P, c) exp-CTD-5P-22P-40P, d) exp-CTD-5P-12P-18P-32P and e) exp-CTD-5P-12P-18P-25P-32P-40P. In addition, Y_1-Y_5 represent a few of the lowest energy conformations of different CTD sequences.

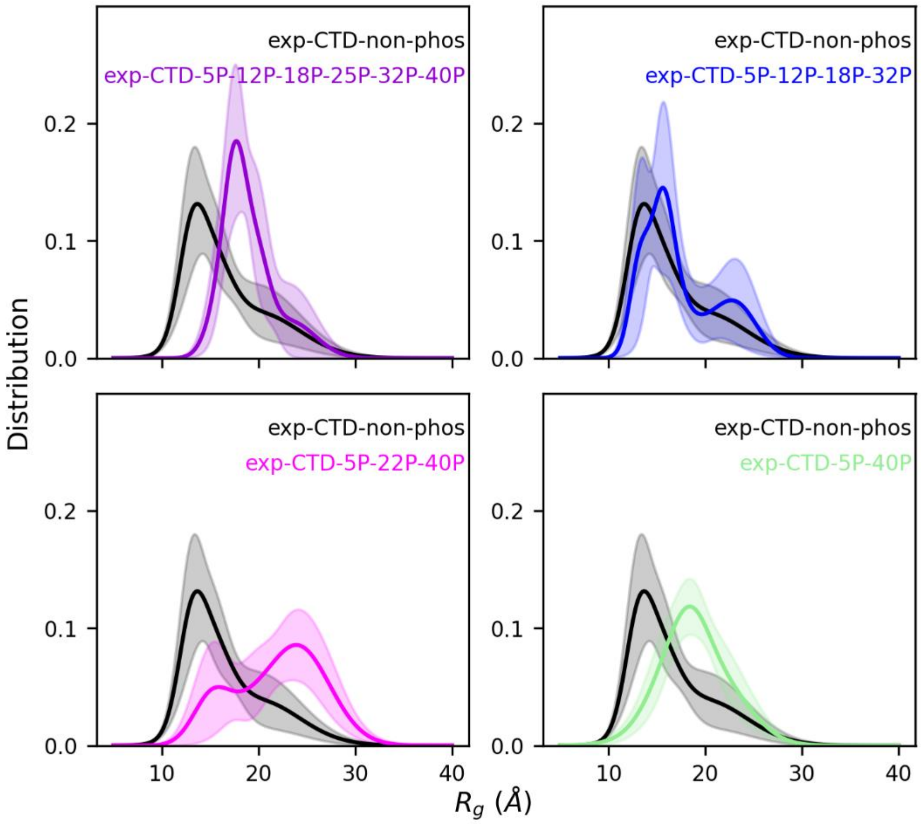

Figure S9: Distribution of radius of gyration (R_g) calculated over the MD simulation trajectories for CTD sequences with 44 residues. The black line shows the R_g distribution for the non-phosphorylated system for comparison. Standard errors are calculated by splitting the full 200 ns trajectory into 40 ns small trajectories.

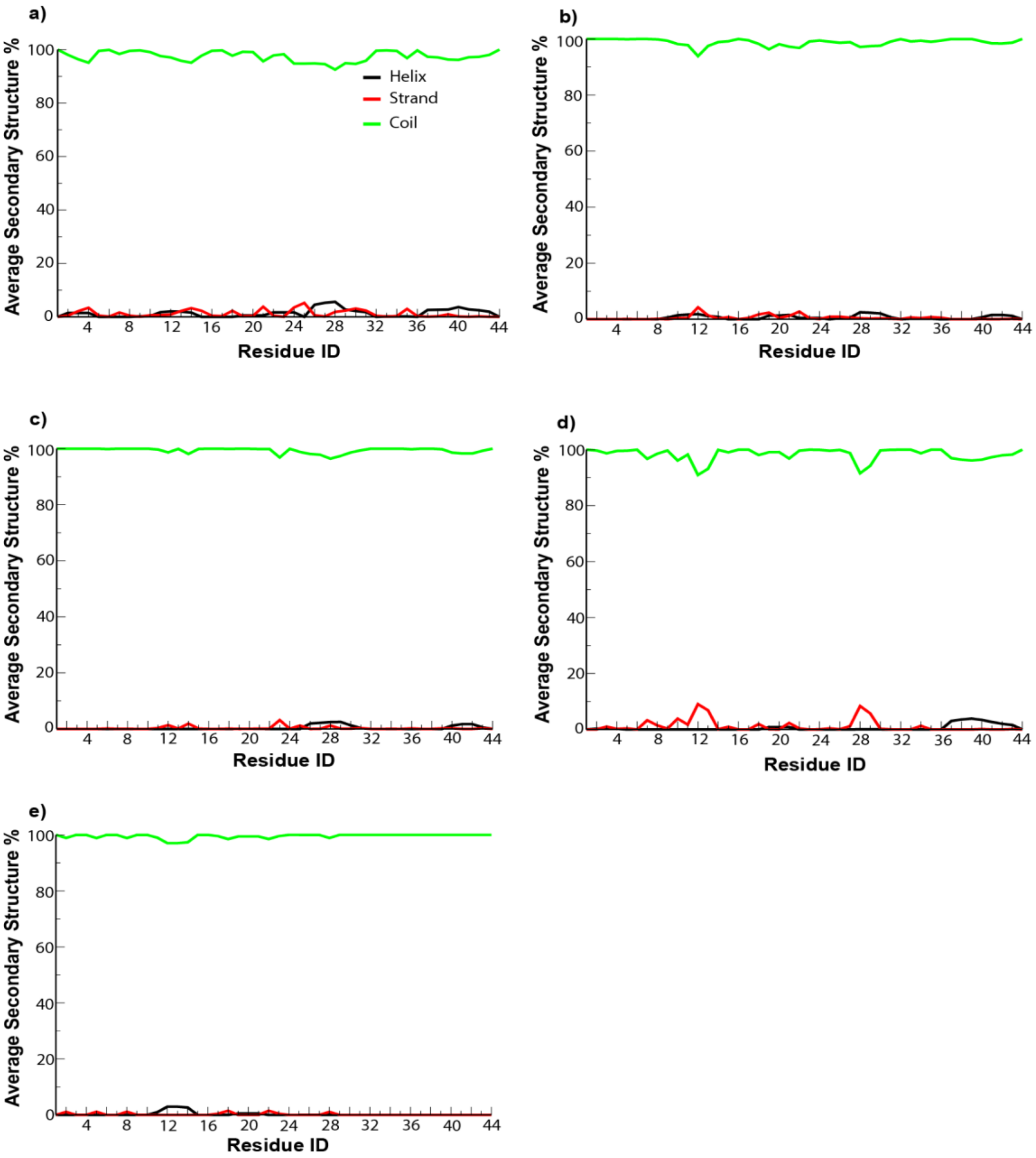

Figure S10: Average secondary structure percentages for CTD sequences with 44 residues. a) exp-CTD-non-phos, b) exp-CTD-5P-40P, c) exp-CTD-5P-22P-40P, d) exp-CTD-5P-12P-18P-32P and e) exp-CTD-5P-12P-18P-25P-32P-40P.

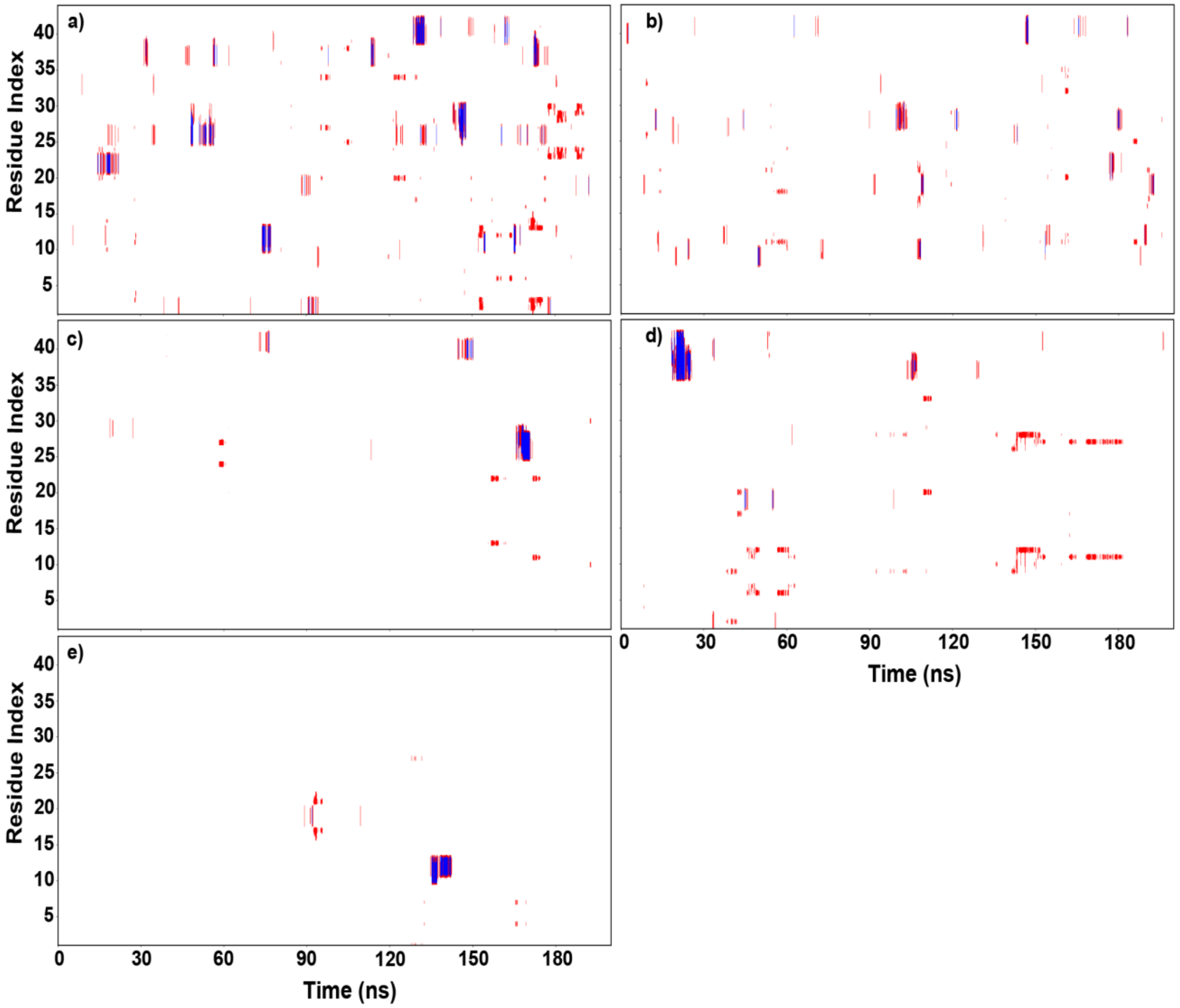

Figure S11: Secondary structure predictions with time for CTD sequences with 44 residues. Coil (loops, bends and turns) structures in white, helix (alpha helix, 3/10 helix and pi helix) structures in blue and strand (beta bridge and extended strand) in red. a) exp-CTD-non-phos, b) exp-CTD-5P-40P, c) exp-CTD-5P-22P-40P, d) exp-CTD-5P-12P-18P-32P and e) exp-CTD-5P-12P-18P-25P-32P-40P.

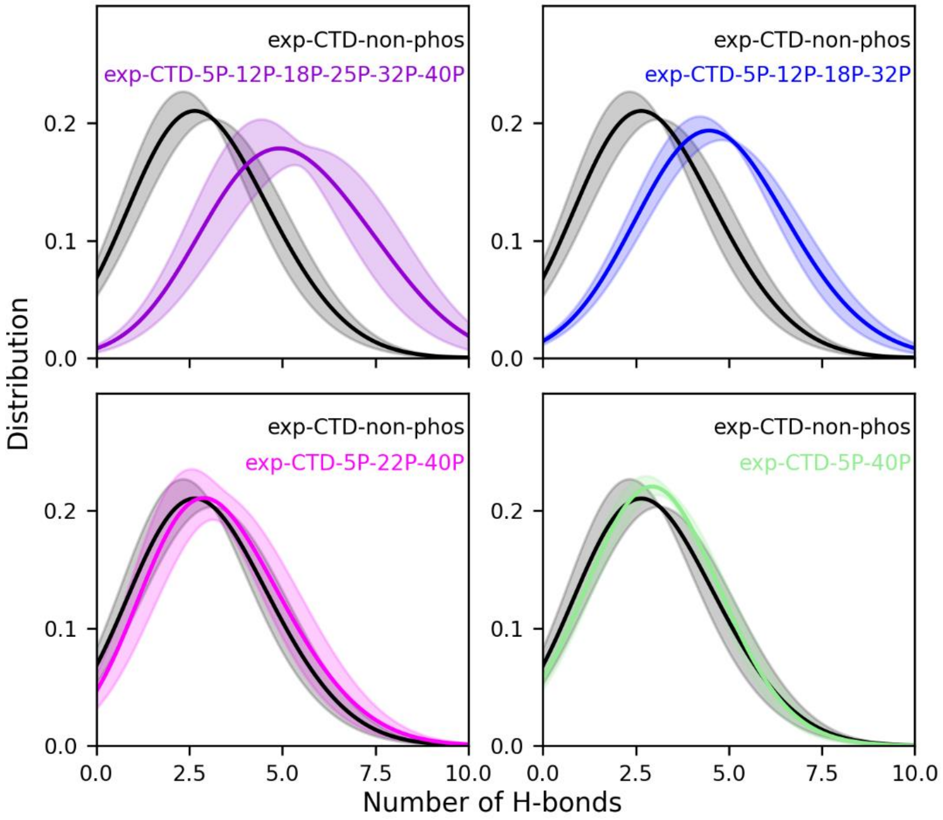

Figure S12: Distribution of total number of intrapeptide H-bonds calculated over the MD simulation trajectories for CTD sequences with 44 residues. The black line shows the H-bond distribution for the non-phosphorylated system for comparison. Error bars are calculated by splitting the full 200 ns trajectory into 40 ns small trajectories.

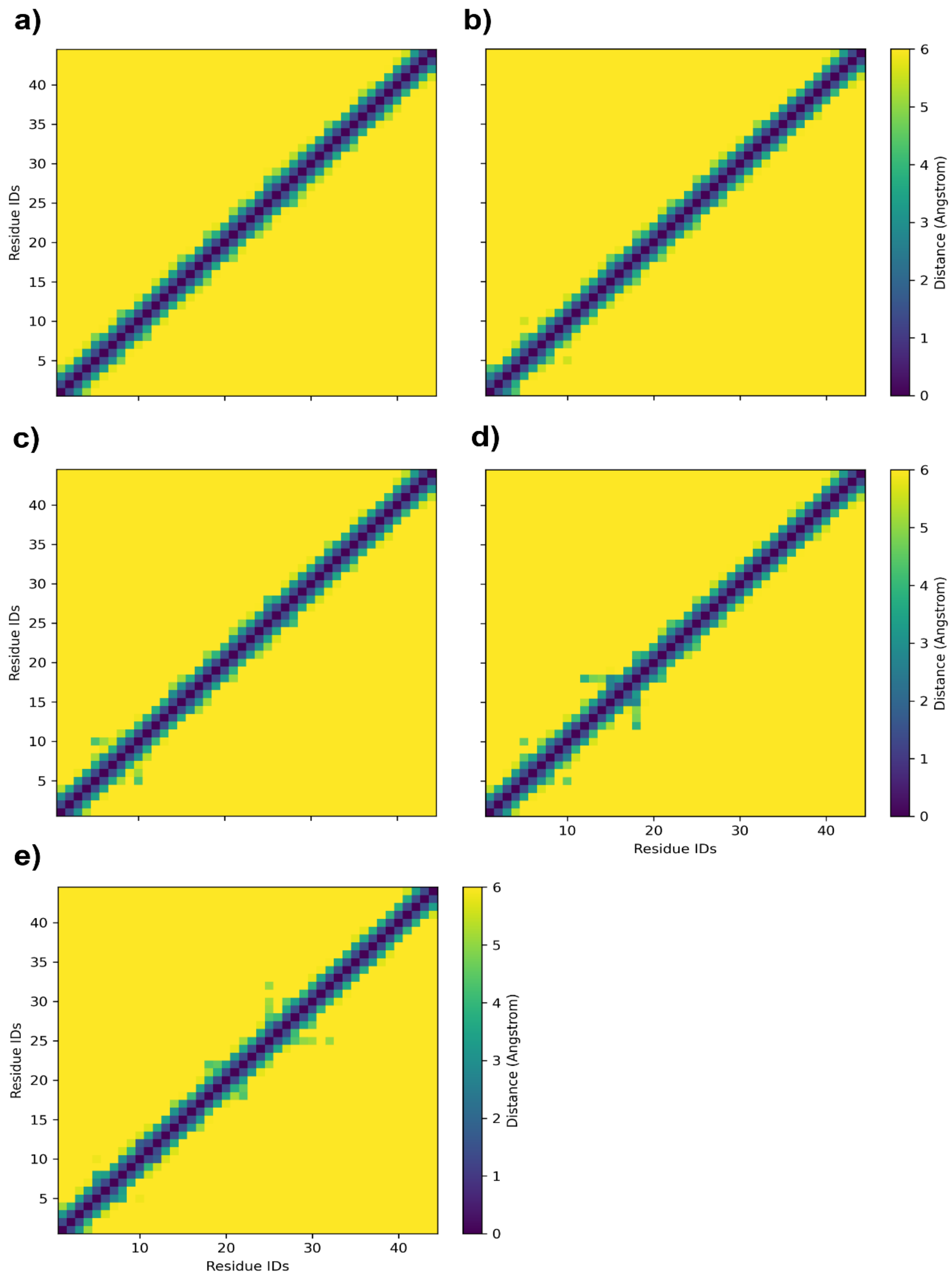

Figure S13: Distance maps generated from the minimum distances between the residues of CTD sequences with 44 residues over the trajectories. a) exp-CTD-non-phos, b) exp-CTD-5P-40P, c) exp-CTD-5P-22P-40P, d) exp-CTD-5P-12P-18P-32P and e) exp-CTD-5P-12P-18P-25P-32P-40P.

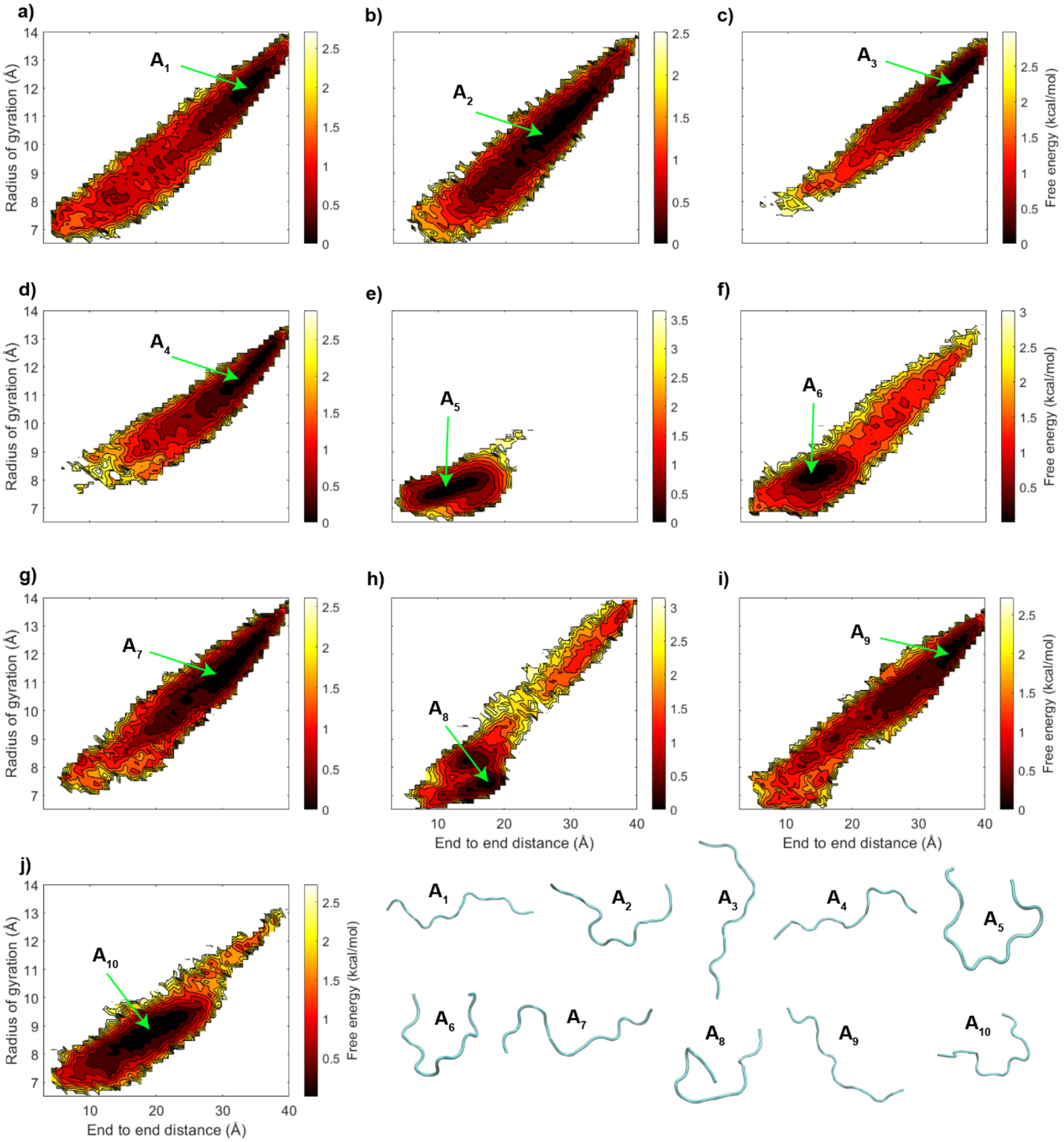

Figure S14: The free energy landscapes using radius of gyration (R_g) and end to end distance as reaction coordinates, a) 2CTD-non-phos, b) 2CTD-2P, c) 2CTD-2P-5P, d) 2CTD-2P-5P-9P, e) 2CTD-2P-5P-9P-12P, f) 2CTD-2P-5P-12P, g) 2CTD-2P-9P, h) 2CTD-2P-12P, i) 2CTD-5P and j) 2CTD-5P-12P. In addition, A_1-A_{10} represents a few of the lowest energy conformations of different CTD sequences.

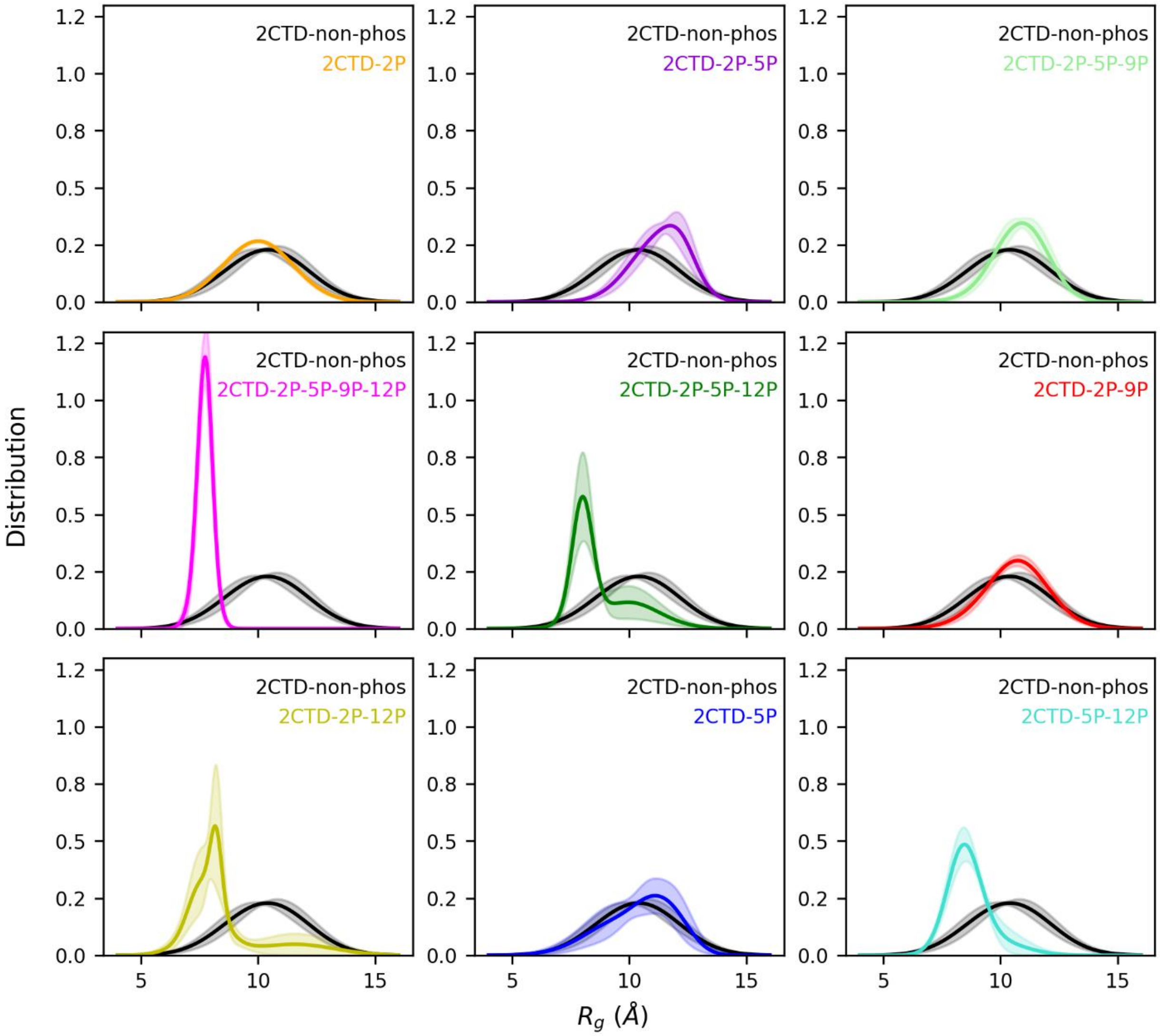

Figure S15: Distribution of radius of gyration (R_g) calculated over the MD simulation trajectories for the 2CTD models. The black line shows the R_g distribution for the non-phosphorylated system for comparison. Error bars are calculated by splitting the full 200 ns trajectory into 40 ns small trajectories, except for 2CTD-2P-5P-9P-12P, which the 400ns trajectory was split into 80 ns small trajectories.

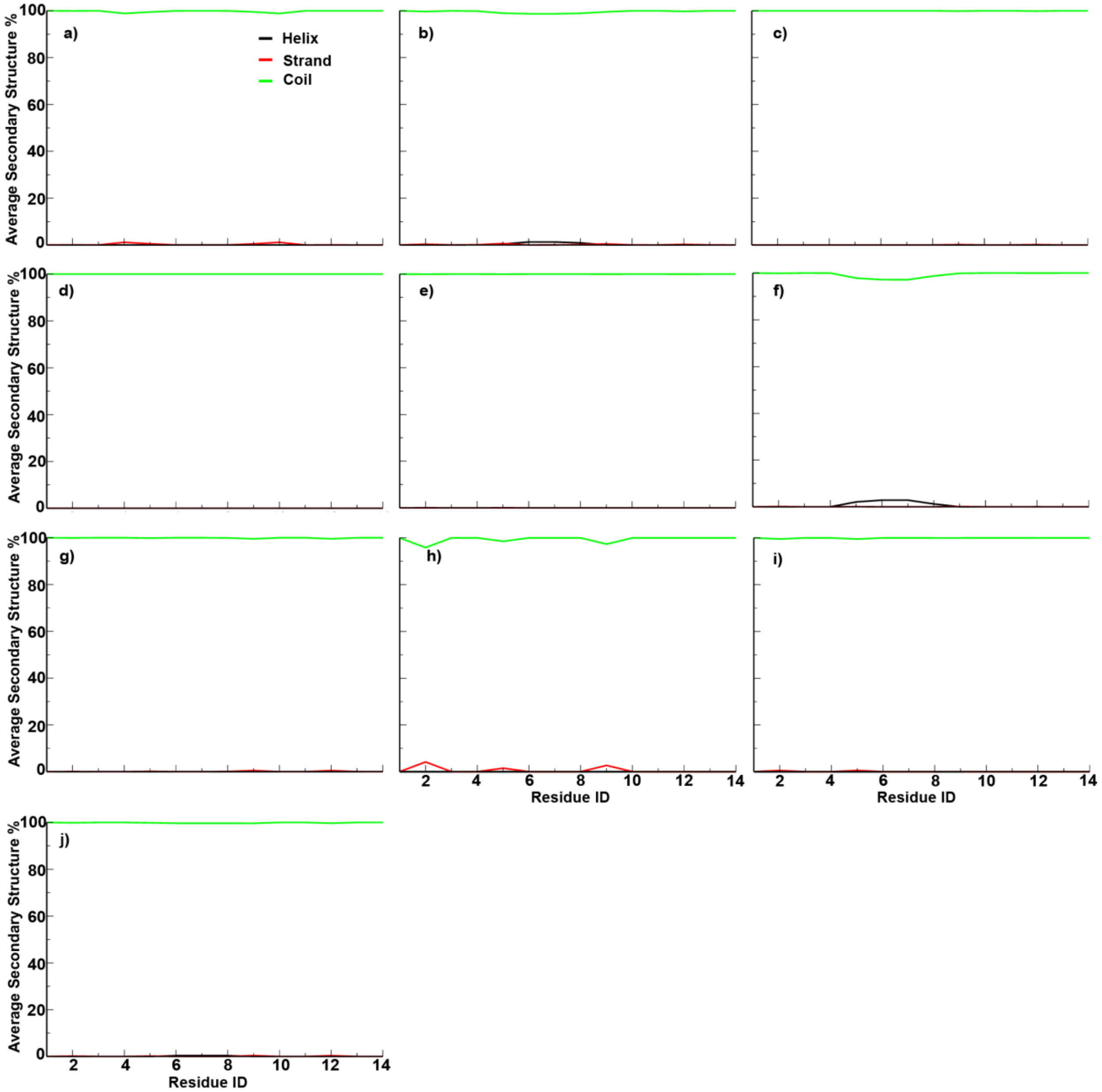

Figure S16: Average secondary structure percentages for CTD sequences with 14 residues. a) 2CTD-non-phos, b) 2CTD-2P, c) 2CTD-2P-5P, d) 2CTD-2P-5P-9P, e) 2CTD-2P-5P-9P-12P, f) 2CTD-2P-5P-12P, g) 2CTD-2P-9P, h) 2CTD-2P-12P, i) 2CTD-5P, j) 2CTD-5P-12P.

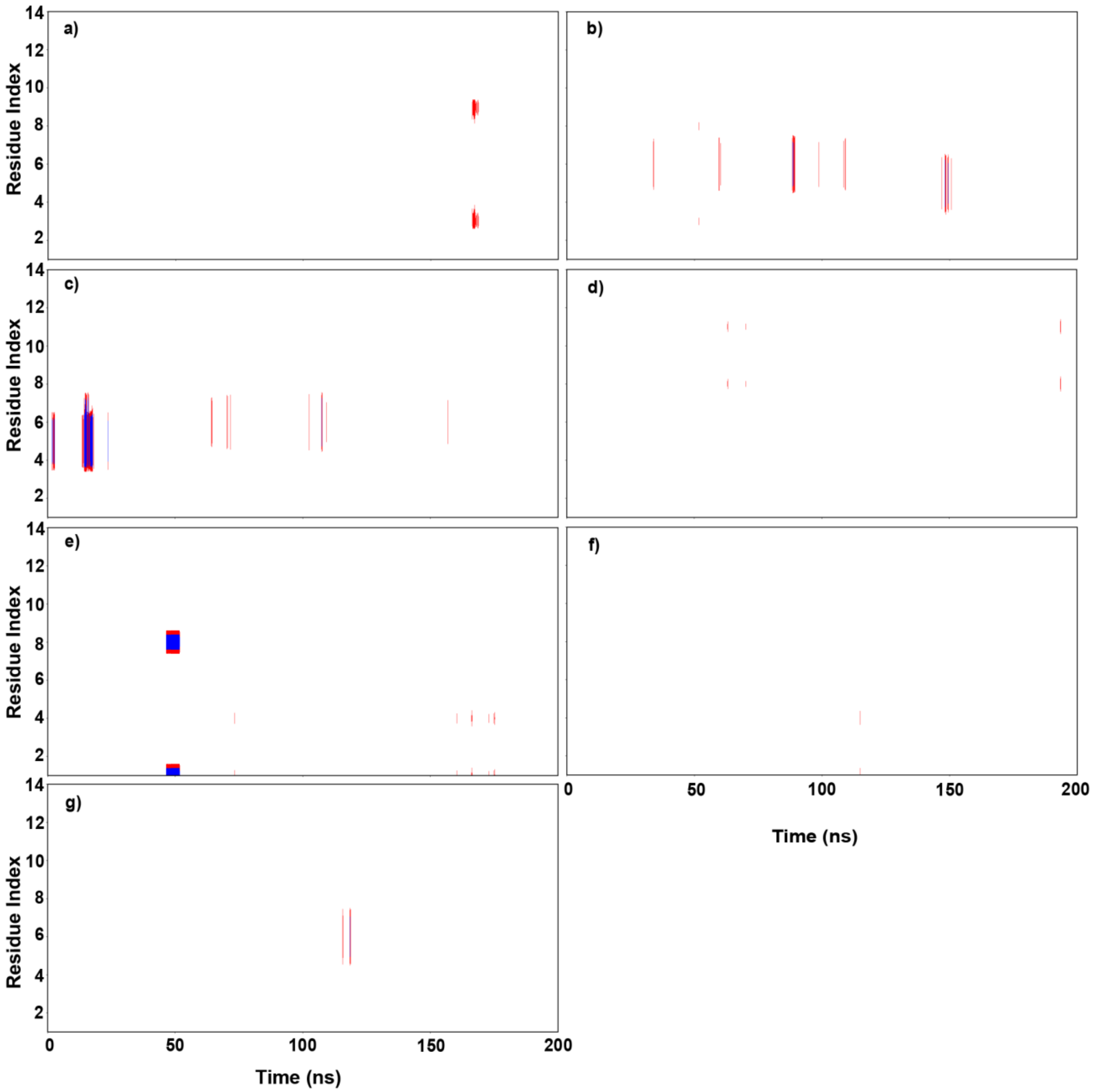

Figure S17: Secondary structure predictions with time for CTD sequences with 14 residues. Coil (loops, bends and turns) structures in white, helix (alpha helix, 3/10 helix and pi helix) structures in blue and strand (beta bridge and extended strand) in red. a) 2CTD-non-phos, b) 2CTD-2P, c) 2CTD-2P-5P-12P, d) 2CTD-2P-9P, e) 2CTD-2P-12P, f) 2CTD-5P and g) 2CTD-5P-12P. Moreover, 2CTD-2P-5P, 2CTD-2P-5P-9P and 2CTD-2P-5P-9P-12P are not shown in this figure because all the secondary structures were coils (loops, turns or bends) for those CTD sequences.

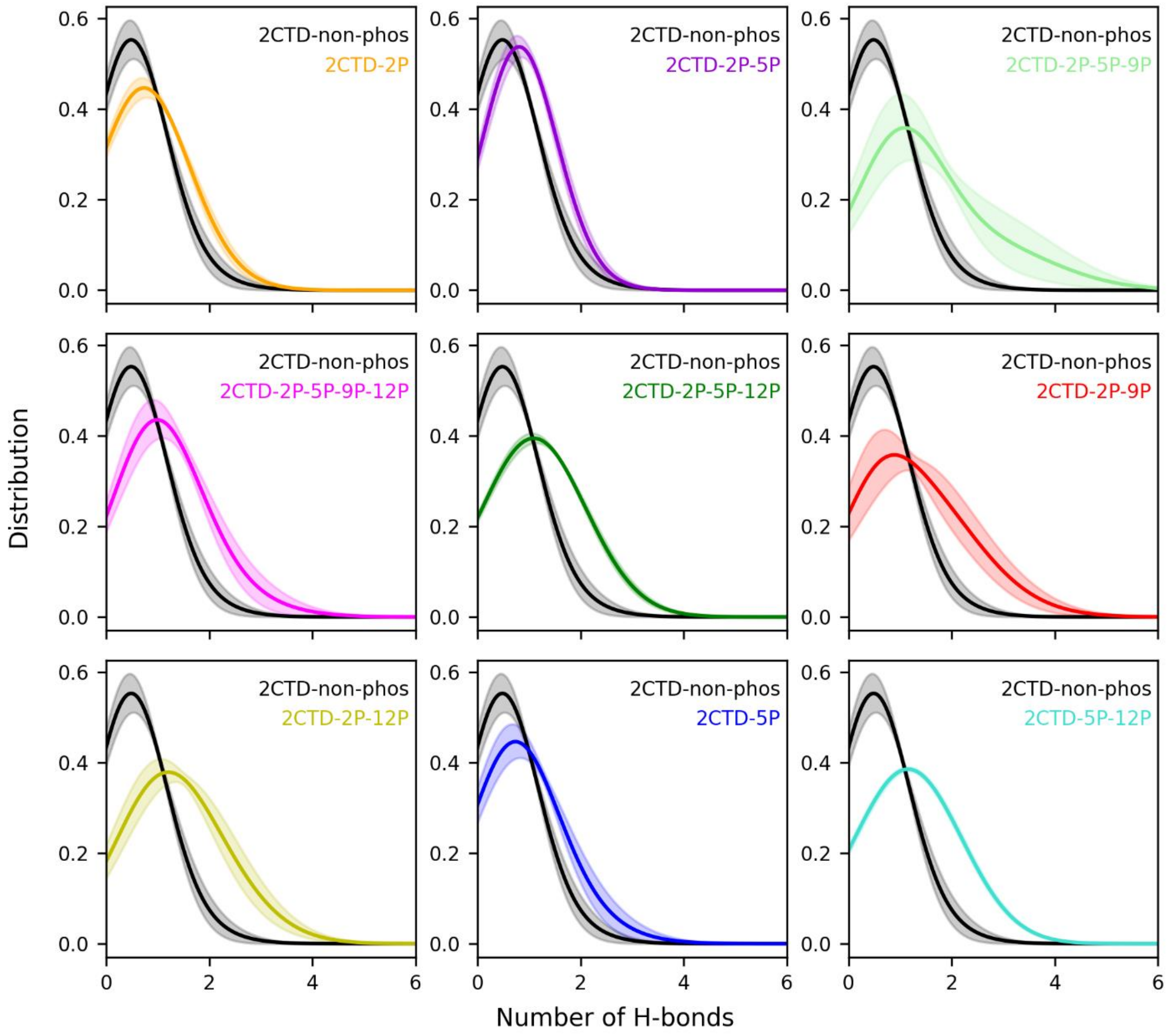

Figure S18: Distribution of total number of intrapeptide H-bonds calculated over the MD simulation trajectories for the 2CTD models. The black line shows the H-bond distribution for the non-phosphorylated system for comparison. Error bars are calculated by splitting the full 200 ns trajectory into 40 ns small trajectories, except for 2CTD-2P-5P-9P-12P, which the 400ns trajectory was split into 80 ns small trajectories.

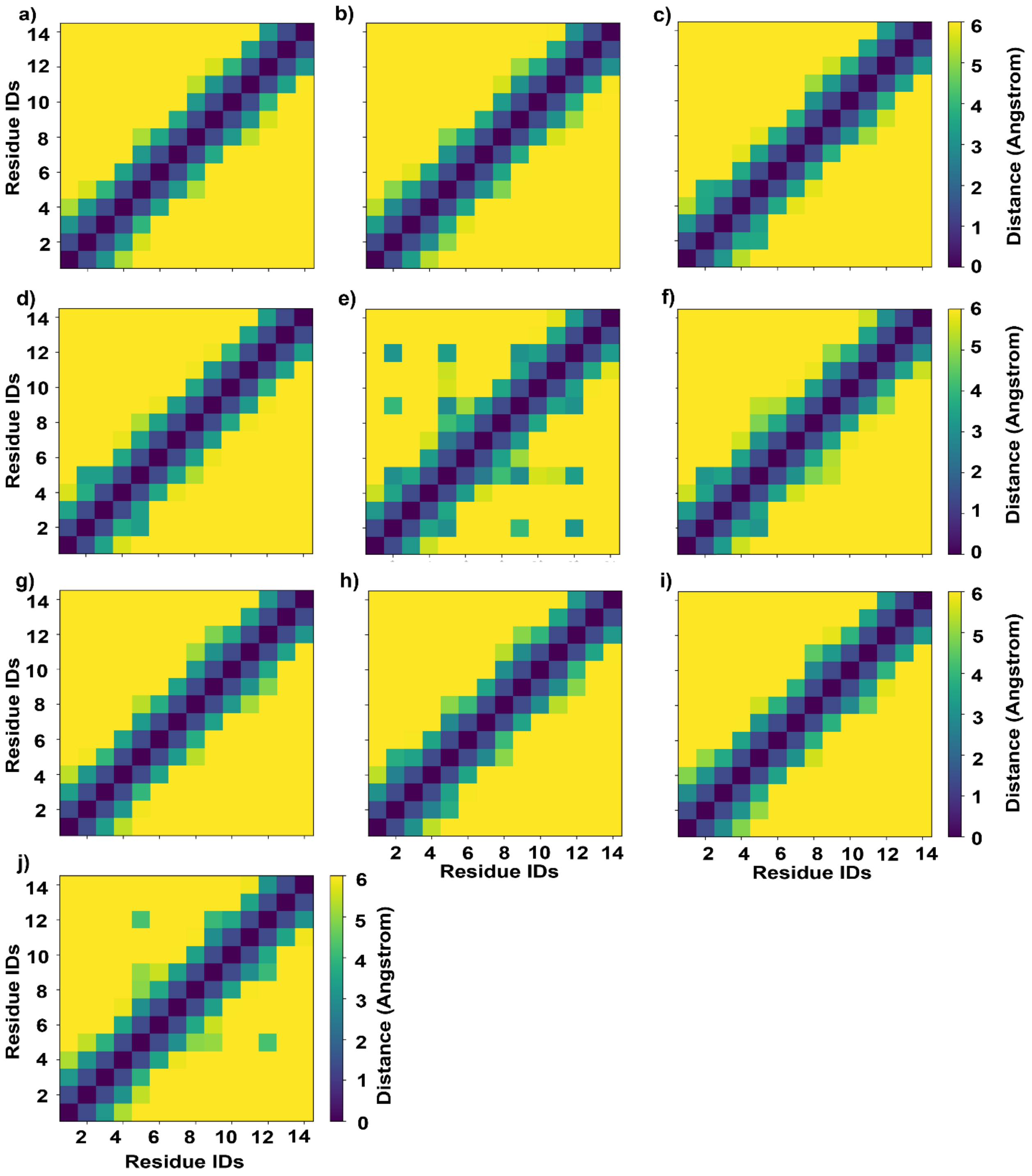

Figure S19: Distance maps generated from the minimum distances between the residues of CTD sequences with 14 residues over the 200 ns trajectories except for 2CTD-2P-5P-9P-12P which the last 400 ns were utilized from full 500 ns trajectory. a) 2CTD-non-phos, b) 2CTD-2P, c) 2CTD-2P-5P, d) 2CTD-2P-5P-9P, e) 2CTD-2P-5P-9P-12P, f) 2CTD-2P-5P-12P, g) 2CTD-2P-9P, h) 2CTD-2P-12P, i) 2CTD-5P and j) 2CTD-5P-12P.

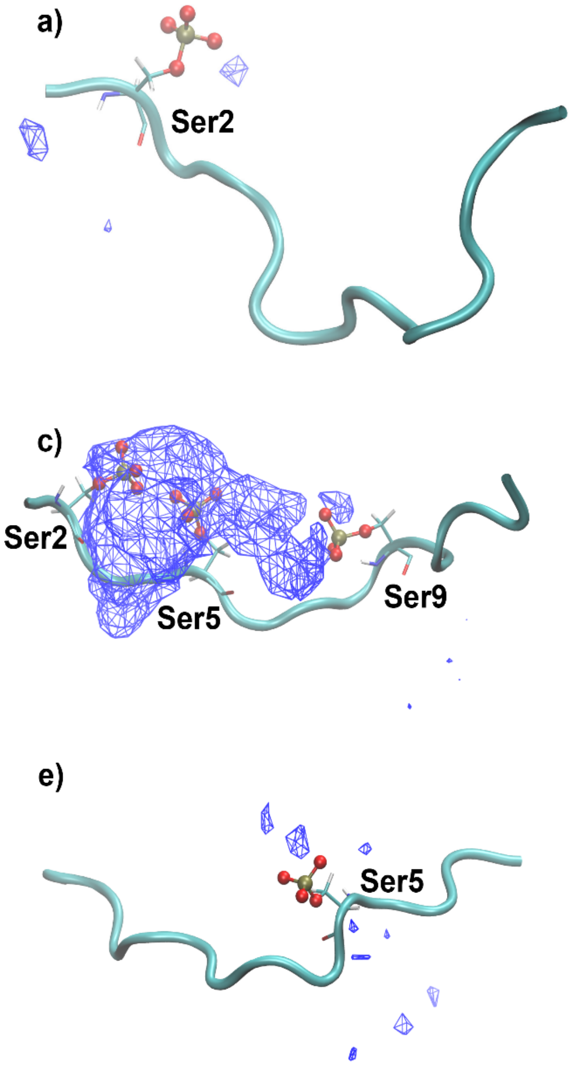

Figure S20: The average Na^+ ion density around the phosphate groups in serine (Ser) residues for the central structures of 2CTDs that did not contract. a) 2CTD-2P, b) 2CTD-2P-5P, c) 2CTD-2P-5P-9P, d) 2CTD-2P-9P and e) 2CTD-5P. The color codes are the same as Figure 6 in the main manuscript.

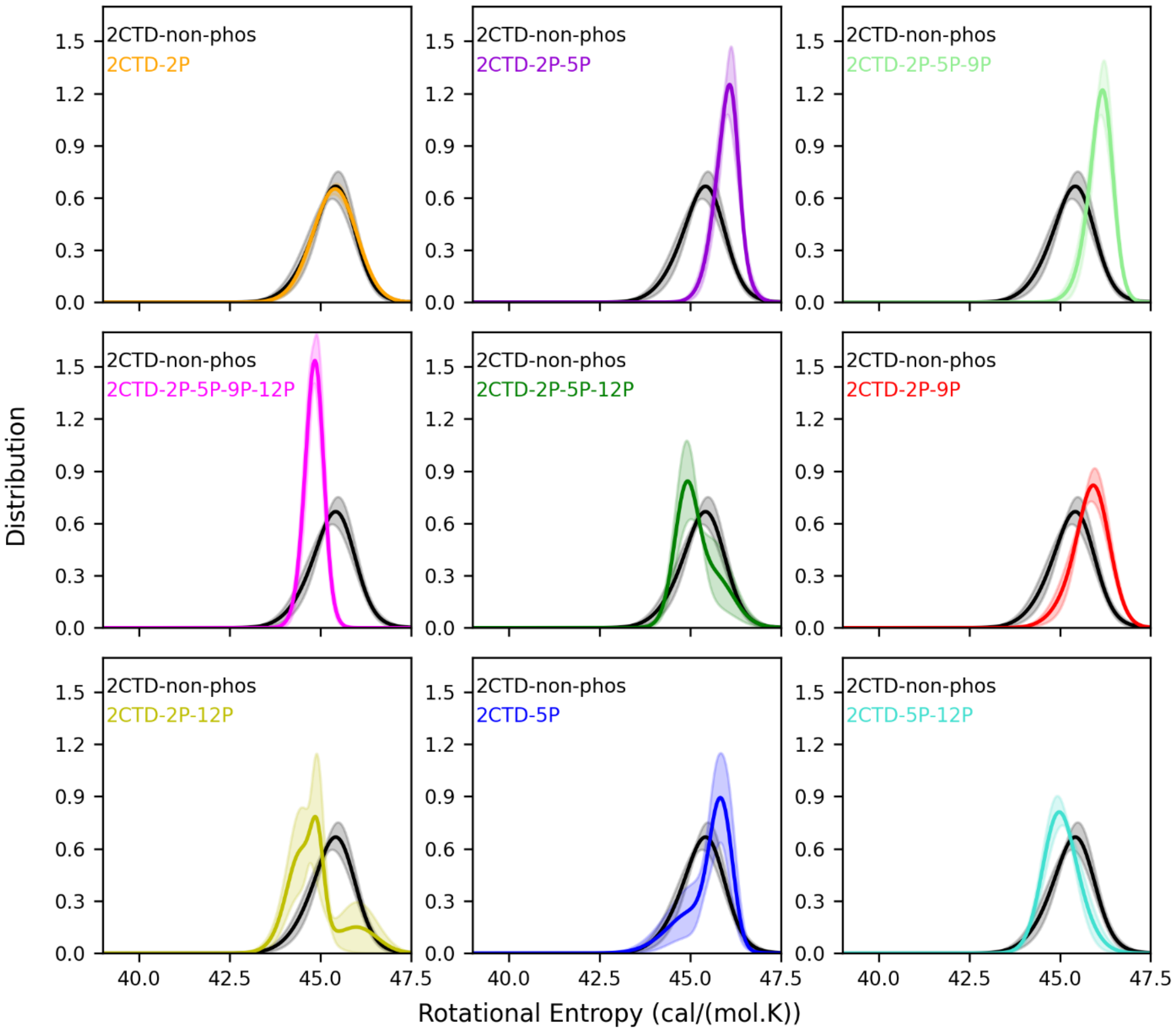

Figure S21: Distribution of rotational entropies calculated over the MD simulation trajectories for 2CTD systems.

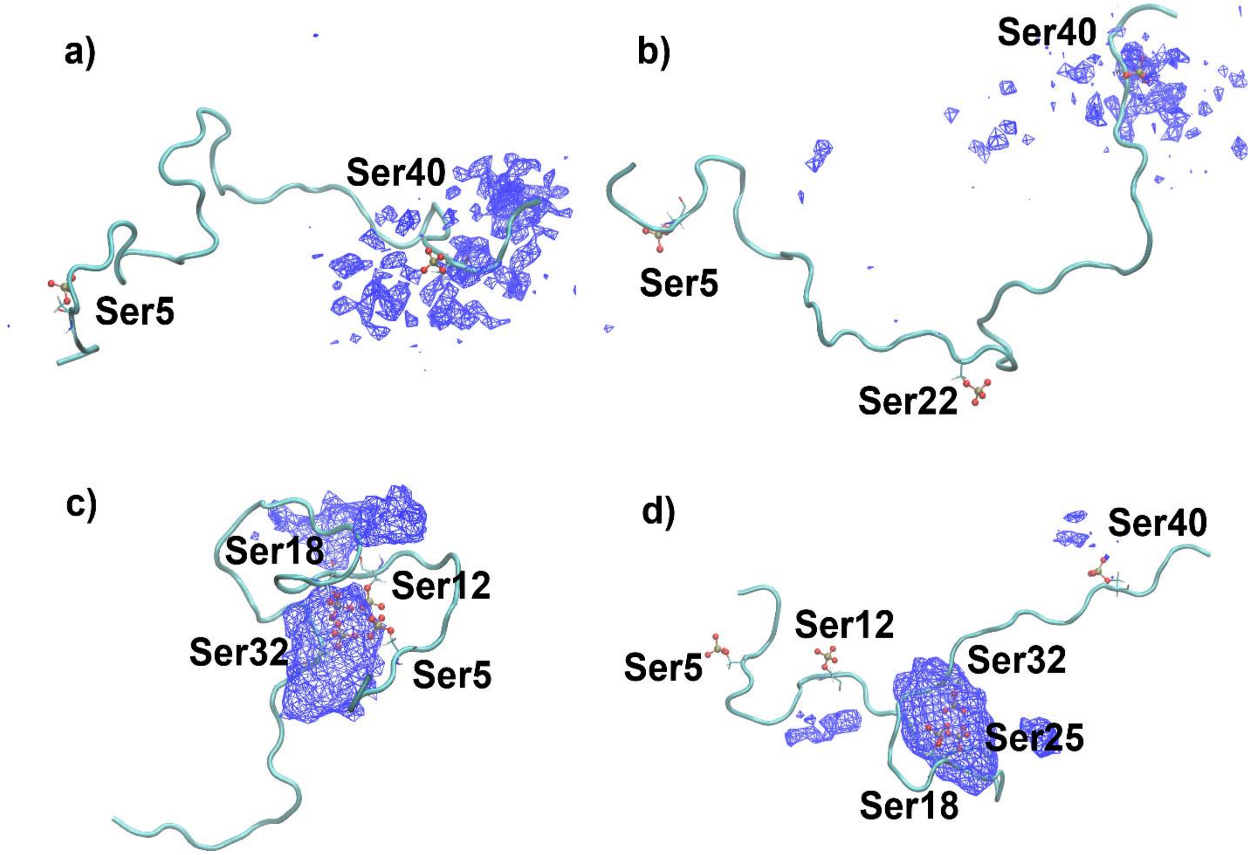

Figure S22: The average Na^{+} ion density around the phosphate groups in serine (Ser) residues for the central structures of exp-CTDs. a) exp-CTD-5P-40P, b) exp-CTD-5P-22P-40P, c) exp-CTD-5P- 12P-18P-32P and d) exp-CTD-5P-12P-18P-25P-32P-40P. The color codes are the same as Figure 6 in the main manuscript.

Table S1: Simulation system details for each CTD model. For each simulation, the cutoff distance from the box edge was set to 10 Å.

System	Total number of atoms	Number of Na^{+} ions	Number of water molecules	Box length (nm)
exp-CTD-non-phos	134702	4	44694	11.2
exp-CTD-5P-40P	134652	8	44674	11.2
exp-CTD-5P-22P-40P	134612	10	44659	11.2
exp-CTD-5P-12P-18P-32P	134623	12	44661	11.2
exp-CTD-5P-12P-18P-25P-32P-40P	134564	16	44638	11.2
2CTD-non-phos	22620	0	7472	6.2
2CTD-2P	22601	2	7464	6.2
2CTD-2P-5P	22591	4	7459	6.2
2CTD-2P-5P-9P	22563	6	7448	6.2
2CTD-2P-5P-9P-12P	22532	8	7436	6.2
2CTD-2P-5P-12P	22563	6	7448	6.2
2CTD-2P-9P	22570	4	7452	6.2
2CTD-2P-12P	22594	4	7460	6.2
2CTD-5P	22592	2	7461	6.2
2CTD-5P-12P	22585	4	7457	6.2

Table S2: Total acceptance ratios between the neighboring replicates for REMD simulations of CTD models with 14 (8 replicates) and 44 (16 replicates) residues.

**System**	**Acceptance ratio (%)**
exp-CTD-non-phos	34.774
exp-CTD-5P-40P	34.772
exp-CTD-5P-22P-40P	34.777
exp-CTD-5P-12P-18P-32P	34.783
exp-CTD-5P-12P-18P-25P-32P-40P	34.783
2CTD-non-phos	36.352
2CTD-2P	36.377
2CTD-2P-5P	36.348
2CTD-2P-5P-9P	36.374
2CTD-2P-5P-9P-12P	36.366
2CTD-2P-5P-12P	36.361
2CTD-2P-9P	36.413
2CTD-2P-12P	36.359
2CTD-5P	36.364
2CTD-5P-12P	36.379

Table S3: P-values from the T-tests of the distributions of radius of gyration and H-bonds for phosphorylated states from both 2CTDs and exp-CTDs with respect to their non-phosphorylated states. If the p-value has a decimal point power which is less than 10^{-10} it was considered as 0.0 for the following table.

System	p-value for R_g	p-value for H-bonds
exp-CTD-5P-40P	0.0	0.17
exp-CTD-5P-22P-40P	0.0	1.46×10^{-6}
exp-CTD-5P-12P-18P-32P	8.74×10^{-7}	0.0
exp-CTD-5P-12P-18P-25P-32P-40P	0.0	0.0
2CTD-2P	5.06×10^{-6}	0.0
2CTD-2P-5P	0.0	0.0
2CTD-2P-5P-9P	0.0	0.0
2CTD-2P-5P-9P-12P	0.0	0.0
2CTD-2P-5P-12P	0.0	0.0
2CTD-2P-9P	1.75×10^{-6}	0.0
2CTD-2P-12P	0.0	0.0
2CTD-5P	0.35	0.0
2CTD-5P-12P	0.0	0.0

Table S4: Average number of intrapeptide H-bonds for CTDs with 44 residues and 14 residues.

System	Average number of H-bonds
exp-CTD-non-phos	2.8793
exp-CTD-5P-40P	2.9900
exp-CTD-5P-22P-40P	3.2640
exp-CTD-5P-12P-18P-32P	4.6228
exp-CTD-5P-12P-18P-25P-32P-40P	5.2206
2CTD-non-phos	0.5363
2CTD-2P	0.7627
2CTD-2P-5P	0.8333
2CTD-2P-5P-9P	1.5281
2CTD-2P-5P-9P-12P	1.1204
2CTD-2P-5P-12P	1.1054
2CTD-2P-9P	1.2132
2CTD-2P-12P	1.3214
2CTD-5P	0.8317
2CTD-5P-12P	1.1543

Table S5: Densities of phosphorylated residues with respect to each sequence. Density_1 represents the number of phosphorylated residues divided by the total number of residues in the sequence while Density_2 represents the number of phosphorylated residues divided by the total number of Serine residues in the sequence.

**System**	**Density_1**	**Density_2**
exp-CTD-5P-40P	0.046	0.167
exp-CTD-5P-22P-40P	0.068	0.25
exp-CTD-5P-12P-18P-32P	0.091	0.333
exp-CTD-5P-12P-18P-25P-32P-40P	0.136	0.5
2CTD-2P	0.071	0.167
2CTD-2P-5P	0.143	0.333
2CTD-2P-5P-9P	0.214	0.5
2CTD-2P-5P-9P-12P	0.286	0.667
2CTD-2P-5P-12P	0.214	0.5
2CTD-2P-9P	0.143	0.333
2CTD-2P-12P	0.143	0.333
2CTD-5P	0.071	0.167
2CTD-5P-12P	0.143	0.333
